# Cost-effectiveness of direct surgery versus preoperative octreotide therapy for growth-hormone secreting pituitary adenomas

**DOI:** 10.1007/s11102-022-01270-8

**Published:** 2022-08-27

**Authors:** Lisa Caulley, Eline Krijkamp, Mary-Anne Doyle, Kednapa Thavorn, Fahad Alkherayf, Nick Sahlollbey, Selina X. Dong, Jason Quinn, Stephanie Johnson-Obaseki, David Schramm, Shaun J. Kilty, Myriam G. M. Hunink

**Affiliations:** 1 Department of Otolaryngology-Head and Neck Surgery, University of Ottawa, Ottawa, ON K1H 8L6 Canada; 2 Clinical Epidemiology Program, The Ottawa Hospital Research Institute, Ottawa, Canada; 3 Department of Epidemiology, Erasmus University Medical Center Rotterdam, Rotterdam, The Netherlands; 4 Department of Medicine, Endocrinology and Metabolism, University of Ottawa, Ottawa, Canada; 5 The Ottawa Hospital Research Institute, Ottawa, Canada; 6 School of Epidemiology and Public Health, University of Ottawa, Ottawa, ON Canada; 7 Department of Neurosurgery, University of Ottawa, Ottawa, Canada; 8 Department of Undergraduate Medicine, University of Ottawa, Ottawa, Canada; 9 Department of Pathology, Dalhousie University, Halifax, Canada; 10 Department of Epidemiology and Biostatistics and Department of Radiology and Nuclear Medicine, Erasmus University Medical Center Rotterdam, Rotterdam, The Netherlands; 11 Center for Health Decision Sciences, Harvard T.H. Chan School of Public Health, Boston, USA

**Keywords:** Growth-hormone, Cost-effectiveness, Pituitary adenomas, Decision model

## Abstract

**Purpose:**

The objective of this study was to compare the cost-effectiveness of preoperative octreotide therapy followed by surgery versus the standard treatment modality for growth-hormone secreting pituitary adenomas, direct surgery (that is, surgery without preoperative treatment) from a public third-party payer perspective.

**Methods:**

We developed an individual-level state-transition microsimulation model to simulate costs and outcomes associated with preoperative octreotide therapy followed by surgery and direct surgery for patients with growth-hormone secreting pituitary adenomas. Transition probabilities, utilities, and costs were estimated from recent published data and discounted by 3% annually over a lifetime time horizon. Model outcomes included lifetime costs [2020 United States (US) Dollars], quality-adjusted life-years (QALYs) and incremental cost-effectiveness ratios (ICERs).

**Results:**

Under base case assumptions, direct surgery was found to be the dominant strategy as it yielded lower costs and greater health effects (QALYs) compared to preoperative octreotide strategy in the second-order Monte Carlo microsimulation. The ICER was most sensitive to probability of remission following primary therapy and duration of preoperative octreotide therapy. Accounting for joint parameter uncertainty, direct surgery had a higher probability of demonstrating a cost-effective profile compared to preoperative octreotide treatment at 77% compared to 23%, respectively.

**Conclusions:**

Using standard benchmarks for cost-effectiveness in the US ($100,000/QALY), preoperative octreotide therapy followed by surgery may not be cost-effective compared to direct surgery for patients with growth-hormone secreting pituitary adenomas but the result is highly sensitive to initial treatment failure and duration of preoperative treatment.

**Supplementary Information:**

The online version contains supplementary material available at 10.1007/s11102-022-01270-8.

## Introduction

Health care costs have continued to increase in high income countries over the last two decades [1, 2]. Current health policy initiatives continue to exacerbate this problem by increasing access to an inefficient system, thus, further driving costs, while failing to address the fundamental problem: an inability to deliver improved patient outcomes at a lower total cost. This has increased the need to identify strategies that improve the relative cost-effectiveness of health care delivery.

Pituitary tumors account for 10 to 15% of all diagnosed intracranial tumors, 90% of which are adenomas [3]. Current prevalence studies suggest that approximately two-thirds of pituitary adenomas are symptomatic due to hypersecretion of hormones [4, 5]. Growth hormone (GH)-secreting pituitary adenoma subtype accounts for approximately 10 to 15% of pituitary adenomas and have been found to be the root cause of 95% of the endocrine disorder, acromegaly [6–10]. A rare but severe and debilitating disease, the deleterious effects of chronic GH excess are associated with mass tumour effects, dysmorphic craniofacial features, cardiovascular, respiratory and metabolic dysfunction, arthropathies, chronic disability and impaired quality of life [7, 11–21]. Elevated morbidity and mortality in acromegalic patients correlates with GH levels and therefore efficient treatment modalities are important to achieve long-term biochemical tumor control, improve quality of life and decrease mortality [12, 17, 20, 22–24]. Surgery, medical therapy, and radiotherapy are the current treatment options available for the management of GH-secreting adenomas, but there is continued dispute regarding their roles. As a result, effective treatment algorithms in the management of treatment-naive and recurrent or residual disease are lacking [14, 25]. Surgery offers immediate lowering of GH levels, and provides tissue for confirmatory histopathologic analysis. Pharmacological therapy is recognized to improve surgical outcomes in selected settings [14]. Similarly, pre-operative pharmacological therapy has been found to result in tumor size reduction and improve surgical remission rates in select prospective studies [26–29]. However, current guidelines do not recommend the routine use of pre-operative pharmacological therapy in the management of GH-secreting pituitary tumors [30]. Nevertheless, multiple clinical trials continue.

The conflicting data in the current literature regarding the best treatment algorithm for GH-secreting adenomas result from the rarity of the disease which restricts the data to underpowered prospective studies and limited follow up. A model-based synthesis of the evidence on the most effective and safe management is needed to guide decision-making and inform the shift towards individualized patient treatment. The purpose of this decision analytic model is to fill this knowledge gap and provide physicians with an evidence-based treatment approach for patients with previously untreated GH-secreting pituitary adenomas from a health-care sector perspective.

## Methods

### Model description

**Fig. 1:**
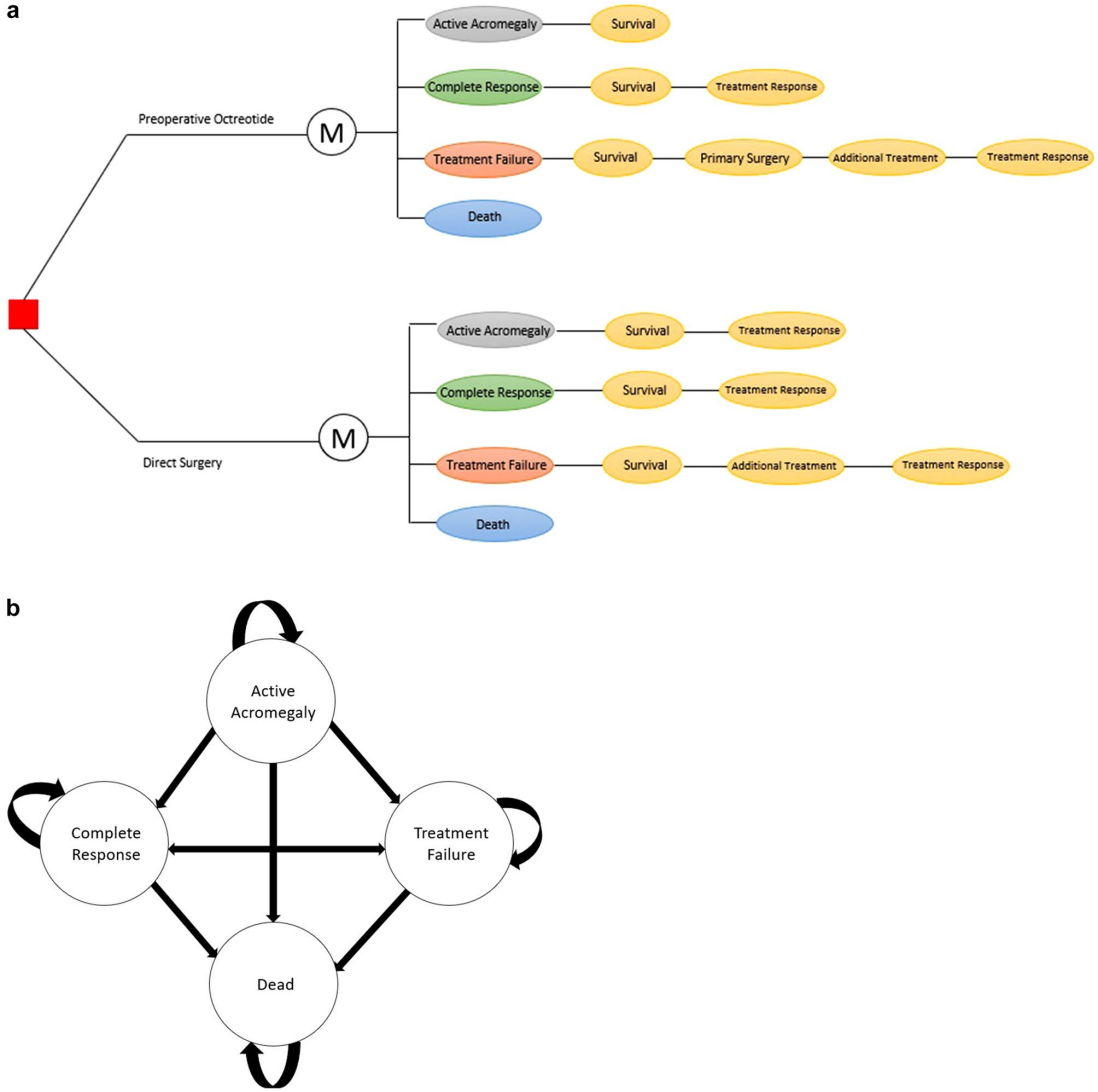
**a** Decision-analytic model schematic representing the health states following primary treatment for growth-hormone secreting pituitary adenomas. **b** Model structure representing the 3-month state-transitions. Survival: Transition to an alive state (that is, complete response or treatment failure health state) or death state. Treatment Response: Transition to complete response or treatment failure health states. Additional treatment: Treatment strategies included revision surgery, gamma-knife radiosurgery, and post-radiation medical therapy

An individual-level state-transition model with four health states was developed to estimate the lifetime health and economic consequences of surgical and medical therapy in patients with previously untreated GH-secreting pituitary adenomas from a health-care sector perspective (Fig. 1). The model compared the current recommended primary treatment approach of direct surgery (that is, surgery without preoperative treatment) to preoperative octreotide treatment and modeled postoperative treatment strategies for residual or persistent tumor-related disease. A hypothetical United States (US) cohort of 100,000 patients with GH-secreting pituitary adenomas was modeled in a microsimulation. As this study involved a simulated cohort of patients and did not involve human participants, ethics approval was not required.

Patients entered the model between age 40 and 47. All patients started in a state of active disease, defined as GH hypersecretion from pituitary tumors that leads to overproduction of insulin-like growth factor1 (IGF-1) and multisystem disease. Following primary treatment, individuals transitioned every 3 months between 4 of the mutually exclusive and collectively exhaustive health states (that is, active acromegaly, complete response to treatment, treatment failure, and death) until biochemical control was achieved for 5 years and then annually until death (Fig. 1). Complete response to treatment was defined as individuals attaining age-normalized IGF-1 levels following treatment, and treatment failure was defined as the inability to achieve a complete response. Patients who experienced treatment failure were assumed to be symptomatic from their persistent disease. Patients who achieved complete response for 5 years were assumed to no longer be at risk for treatment failure.

### Treatment strategies

The treatment strategy “preoperative octreotide” therapy involved 6 months of octreotide treatment. [31]. Patients who were responsive to medical therapy at 6 months could continue to be observed on octreotide to maintain biochemical remission [32]. Patients who failed to achieve biochemical remission with 6 months of octreotide underwent transsphenoidal surgery.

Direct surgery was defined as endoscopic endonasal transsphenoidal surgery performed by a neurosurgeon and otolaryngologist-head and neck surgeon. Patients were assumed to undergo three nasal debridements within 6 months of surgery. Patients that did not achieve biochemical remission within 3 months of surgery received treatment for recurrent or persistent disease. The sequence of treatment for recurrent or persistent disease were identical for both treatment strategies [14, 33]. Patients received octreotide therapy for a minimum of 6 months. Patients could then continue on 3-month courses of octreotide for persistent or recurrent disease. Patients who did not achieve biochemical cure on the highest dose of octreotide within 18 months received cabergoline in addition to octreotide for 12 months [34, 35]. Patients that were refractory to combination octreotide and cabergoline were transitioned to cabergoline and pegvisomant for 6 months [36]. Patients that failed post-surgery medical therapy were eligible for revision surgery, adjunctive radiotherapy (that is, gamma-knife radiosurgery), followed by additional salvage medical therapy (that is, temozolomide) [14, 30, 37, 38]. Gamma-knife radiosurgery was assumed to deliver highly focused ionizing beams to residual or recurrent disease in a single session. Patients received salvage therapy for a maximum of 24 months before assumed remission. Complications that occurred following surgery, medical therapy, or radiation therapy were assumed be treated within 3 months.

### Clinical parameters

Health state transition probabilities of moving between health states were based on a systematic review of surgical and non-surgical therapy for GH-secreting adenomas that has been published elsewhere [39]. Transition probabilities were presented per cycle length (that is, 3 month probabilities). Hazard rates were modeled from published RCTs to incorporate time-dependent risk of treatment failure after surgery alone, and preoperative medical and surgical therapy (eFig. 1) [39]. Observational studies suggest that rates of long-term remission for primary surgery are similar to those of revision surgery [40, 41]. Effects of stereotactic radiosurgery for residual or relapsed disease were modeled [42, 43]. The transition probabilities used in the model can be found in Table 1.

**Table 1:** Model input parameters

Parameter	Base case estimates	Distribution	References
Age at treatment initiation (years)^a^	40−47	Uniform	[80]
Standardized mortality rate for patients with untreated acromegaly	2.5	Lognormal (2.5, SD: 0.75)	[23]
Intervention			
Surgery alone: probability of treatment failure at 3-month	0.77	Beta (0.77 SD: 0.23)	[26, 81]
Octreotide therapy alone: probability of treatment failure at 6-month	0.78	Beta (0.78; SD: 0.23)	[82]
Preoperative octroetide therapy and surgery^b^: probability of treatment failure at 3-month	0.55	Beta (0.55, SD: 0.17)	[26]
Revision surgery: probability of treatment failure at 3-month	0.43	Beta (0.43, SD: 0.13)	[83]
Postoperative gamma-knife radiosurgery: probability of treatment failure at 3-month	0.45	Beta (0.45, SD: 0.14)	[84]
Post-operative octreotide therapy: probability of 3-month treatment failure	0.18	Beta (0.18, SD: 0.06)	[34]
Post-operative octreotide and cabergoline therapy: probability of 6-month treatment failure	0.20	Beta (0.24, SD: 0.06)	[35]
Post-operative adjuvant pegvisomant and cabergoline therapy: probability of 3-month treatment failure	0.02	Beta (0.02, SD: 0.005)	[85]
Post-radiation temozolomide therapy: probability of treatment failure at 3-month	0.66	Beta (0.66, SD: 0.20)	[86]
Complications: probability of cerebrospinal fluid leak after surgery at 3 months	0.38	Beta (0.38, SD: 0.11)	[28, 52]
Odds ratio of cerebrospinal fluid leak after preoperative octreotide therapy compared to direct surgery	0.21	Lognormal (0.21, SD: 0.06)	[28, 52]
Complications: probability of diabetes insipidus limited to first 3 months after surgery (transient)	0.026	Beta (0.026, SD: 0.008)	[26, 52]
Odds ratio of diabetes insipidus after preoperative octreotide therapy compared to direct surgery	2.47	Lognormal (2.47, SD: 0.74)	[26, 52]
Complications: probability of persistent diabetes insipidus after surgery at 3 months (persistent)	0.02	Beta (0.02, SD: 0.006)	[87]
Complications: probability of vision impairment after surgery at 3 months	0.007	Beta (0.007, SD: 0.002)	[87]
Complications: probability of hematoma after surgery at 3 months	0.02	Beta (0.02, SD: 0.005)	[87]
Complications: probability of hypopituitarism after surgery at 3 months	0.09	Beta (0.09, SD: 0.03)	[88]
Adverse events of octreotide: probability of biliary problems at 3 months	0.14	Beta (0.14, SD: 0.001)	[89]
Complications: probability of vison impairment and chronic otitis media from gamma-knife radiosurgery at 3 months	0.04	Beta (0.04, SD: 0.0004)	[90]
Utility weights for health states			
Baseline acromegaly	0.70	Beta (0.70, SD: 0.30)	[50, 91]
Complete remission	0.93	Beta (0.93, SD: 0.10)	[92]
Treatment failure	0.70	Beta (0.70, SD: 0.30)	[50]
Death	0	–	–
Complications: cerebrospinal fluid leak after surgery	− 0.03	Lognormal (0.03, SD: 0.009)	[93]
Complications: diabetes insipidus after surgery for 3 months	− 0.05	Lognormal (0.05, SD: 0.02)	[93]
Complications: hematoma after surgery	− 0.41	Lognormal (0.41, SD: 0.12)	[93]
Complications: hypopituitarism after surgery	− 0.07	Lognormal (0.07, SD: 0.02)	[93]
Complications: vision impairment after surgery for 3 months	− 0.03	Lognormal (0.03, SD: 0.009)	[93]
Complications: biliary problems following octreotide therapy alone for 3 months	− 0.01	Lognormal (0.01, SD: 0.003)	[93]
Complications: vision impairment and chronic otitis after adjuvant radiosurgery for 3 months	− 0.06	Lognormal (0.06, SD: 0.02)	[93]
Costs			
Transsphenoidal surgery	$13,022	Gamma ($13,022, SD: $3,907)	[47, 94]
Medical therapy: 3 months of octreotide	$17,092	Gamma ($20,097, SD: $5,128)	[95]
Medical therapy: 3 months of pegvismonant	$15,735	Gamma ($15,735, SD: $4,721)	[96]
Adjuvant gamma knife radiosurgery	$13,739	Gamma ($13,931, SD: $4,122)	[90]
Salvage therapy: 3 months of temozolomide	$8,446	Gamma ($8,446, SD: $2,534)	[97]
Complications: 3 months of biliary problems following medical therapy	$168	Gamma ($168, SD: $50)	[95]
Complication: cerebrospinal fluid leak after surgery	$18,804	Gamma ($18,804, SD: $5,641)	[88]
Complications: diabetes insipidus limited to first 3 months after surgery (transient)	$128	Gamma ($128, SD: $38)	[88]
Complications: 3 months of persistent diabetes insipidus	$480	Gamma ($480, SD: $144)	[88]
Complications: hematoma after surgery	$22,489	Gamma ($22,489, SD: $6,747)	[88]
Complications: 3 months of hypopituitarism after surgery	$555	Gamma ($555, SD: $167)	[88]
Complications: 3 months of vision impairment after surgery	$168	Gamma ($168, SD: $50)	[88]
Complication: 3 months of vision impairment and chronic otitis after adjuvant radiosurgery	$669	Gamma ($669, SD: $201)	[47]
Discount rate	3%	Triangle (Min: 0%, Max: 6%)	[54]

Biochemical remission is reported at 3 months post-operatively

*IGF-1* insulin-like growth factor 1, *SD* standard deviation

^a^Individuals were assigned an age at initiation of treatment between age 40 and 47 years using a uniform distribution

^b^Individuals received 6 months of octreotide prior to surgery

Age-specific all-cause mortality was derived from the National Vitals Statistics Report for the general US population [44]. The model accounted for the increased standardized mortality rate associated with GH-secreting adenomas [23]. Acromegaly is associated with a two-fold increase in mortality, compared to healthy people primarily due to the associated cardiovascular disease [20, 23, 45, 46]. The increased risk of mortality was modeled in all patients that demonstrated elevated levels of IGF-1 [23]. Mortality in this patient population returned to that of the general population once they achieved biochemical normalisation [17, 20, 22, 23].

### Cost parameters and health utility values

Medical costs were obtained from a public third-party payer perspective. Intervention costs and utilities were derived from the reviewed literature. Costs for surgery were calculated assuming transsphenoidal surgery required 5 h. Patients were assumed to stay in hospital for a total of 4 days and undergo three nasal debridements within 6 months post-operatively [26, 47, 48]. The costs included in the model were converted and/or inflated to 2020 US dollars per the Consumer Price Index, All Urban Consumers [49]. Utilities were derived from a population of acromegaly patients [50]. Complications that occurred following surgery, medical therapy or radiation therapy were assumed be treated within 3 months and each event was modeled as a one-time cost and dis-utility [26, 28, 48, 51, 52]. Costs and utilities used in the model were calculated per cycle length (that is, 3 months) and are presented in Table 1.

### Model validation

We performed external validation of the model results by comparing the predicted remission rates and required adjuvant therapy in patients with surgical intervention alone to population-based data from The Ottawa Hospital from 2007 to 2016 and the available literature [28, 53].

### Analysis

The incremental cost-effectiveness ratio (ICER), defined as the additional cost for each additional unit of health benefit (QALYs gained), was used as an efficiency metric. The commonly accepted willingness-to-pay (WTP) threshold of $100,000 United States Dollars (USD) was selected to signify the amount below which an intervention would be considered cost-effective. A health care sector perspective was adopted and costs and health benefits were discounted by 3% per year [54]. A half-cycle correction was built into the analysis to account for the fact that events and transitions could occur at any point during the cycle.

### Sensitivity and scenario analyses

The effects of parameter uncertainty and alternative assumptions on the estimated results were evaluated through a series of one-way sensitivity analyses, where only one model parameter was varied while all others were held constant. We varied salient parameters across ranges reported in the literature. Where ranges were not reported in the literature, a plausible range was established by taking 30% above and below the base estimates. Relevant clinical scenarios were also explored. Scenario analyses were performed to evaluate the role of different radiation therapy modalities including external beam radiation, targeted therapy of macroadenomas as compared to microadenomas, and decreased duration of preoperative octreotide therapy. An exploratory analysis of second-line pasireotide therapy [51, 55, 56] and combination pegvisomant and octreotide therapy [57] were performed to evaluate the role of adjuvant therapies on study results.

To understand the impact of multivariable uncertainty on the cost-effectiveness results, we conducted a probabilistic sensitivity analysis (PSA) with 1,000,000 iterations to estimate the probability that each therapy is cost-effective for a given WTP threshold. Base case mean parameter values were replaced by probability distributions that were informed by the shape of the data, the type of parameter, and the plausible range of values specified in Table 1. Beta distributions were used for utilities and probabilities, gamma distributions for costs and lognormal distributions for comparison statistics (that is, odds ratios and standardized mortality ratio) and dis-utilities.

### Value of information

A value of information analysis is a quantitative method of valuing the expected gain from reducing uncertainty in the model parameters [58–64]. These analyses can help quantify the value of selecting an immediate decision, based on the best available evidence, as compared to a delayed decision in favor of additional research to establish more accurate parameter estimates. An expected value of perfect information (EVPI) analysis was performed to quantify the value of eliminating uncertainty from all model parameters. This EVPI establishes an upper bound on expected benefit of performing a study that would allow us to know the values of all the input parameters with complete certainty. The population EVPI is calculated by multiplying the per patient EVPI by the estimated number of patients over the effective lifetime of the treatment options included in the decision problem [65, 66]. If the EVPI indicated further studies could be worthwhile, we also quantified the value of further research that would eliminate the uncertainty in a limited number of key parameters with the expected value of partial perfect information (EVPPI) and the value of reducing uncertainty in parameter values by estimating the expected value of sample information (EVSI) [67].

The model was programmed and analyses performed in R Studio Version 1.4.1106. (R Project for Statistical Computing) and the coding template from the Decision Analysis in R for Technologies in Health (DARTH) workgroup was used. [68, 69]. This economic evaluation was reported according to the 2022 Consolidated Health Economic Evaluation Reporting Standards (CHEERS) guidelines [70].

## Results

### Model validation

In the simulated population receiving standard therapy (that is, direct surgery), biochemical remission was achieved in 69.3% of patients (eTable 1). In this treatment strategy, 33.5% went on to receive revision surgery and 31.5% received postoperative radiation therapy after they failed to achieve remission on postoperative medical therapy (eTable 1).

### Base case analysis

The distribution of GH-secreting pituitary adenoma patients across lifetime horizon in the Markov model is illustrated in eFig. 2. In the primary analysis, direct surgery yielded an average of 19.64 QALYs at a cost of $ 179,796 in the first-order Monte Carlo microsimulation (Table 2). The strategy of preoperative octreotide strategy yielded an additional 0.04 QALYs at an additional cost of $17,011 per individual over their remaining lifetime. At a WTP threshold of $100,000 per QALY, the preoperative octreotide strategy was not cost-effective strategy with an ICER of $455,747/QALY. Accounting for uncertainty in model parameter estimates, direct surgery was found to be the dominant strategy as it yielded lower costs and greater health effects (QALYs) compared to preoperative octreotide strategy in the second-order Monte Carlo microsimulation (Table 3).

**Table 2:** Results of first-order Monte Carlo simulation of 100,000 patients (discounted)

Modality	Mean costs ($, SD)	Incremental cost ($, SD)	Mean QALYs (SD)	Incremental QALYs (SD)	ICER ($/QALY gained)	Net monetary benefit ($)
Direct surgery	180,234 (96,228)	–	19.41 (5.28)		–	1,760,766
Preoperative octreotide + surgery	197,245 (106,691)	17,011 (33.08)	19.45 (5.25)	0.04 (0.00007)	455,747	1,747,755

*SD* standard deviation

**Table 3:** Results of second-order Monte Carlo microsimulation of 1000 patients and 1000 iterations (discounted)

Modality	Mean costs ($, SD)	Incremental cost ($, SD)	Mean QALYs (SD)	Incremental QALYs (SD)	ICER ($/QALY gained)	Net monetary benefit ($)
Direct surgery	179,795 (48,213)	–	19.64 (2.00)		–	1,784,205
Preoperative octreotide + surgery	197,977 (52,871)	–	19.57 (1.99)	–	Dominated	1,759,023

*SD* standard deviation, *NA* not applicable

### Sensitivity analysis

**Fig. 2:**
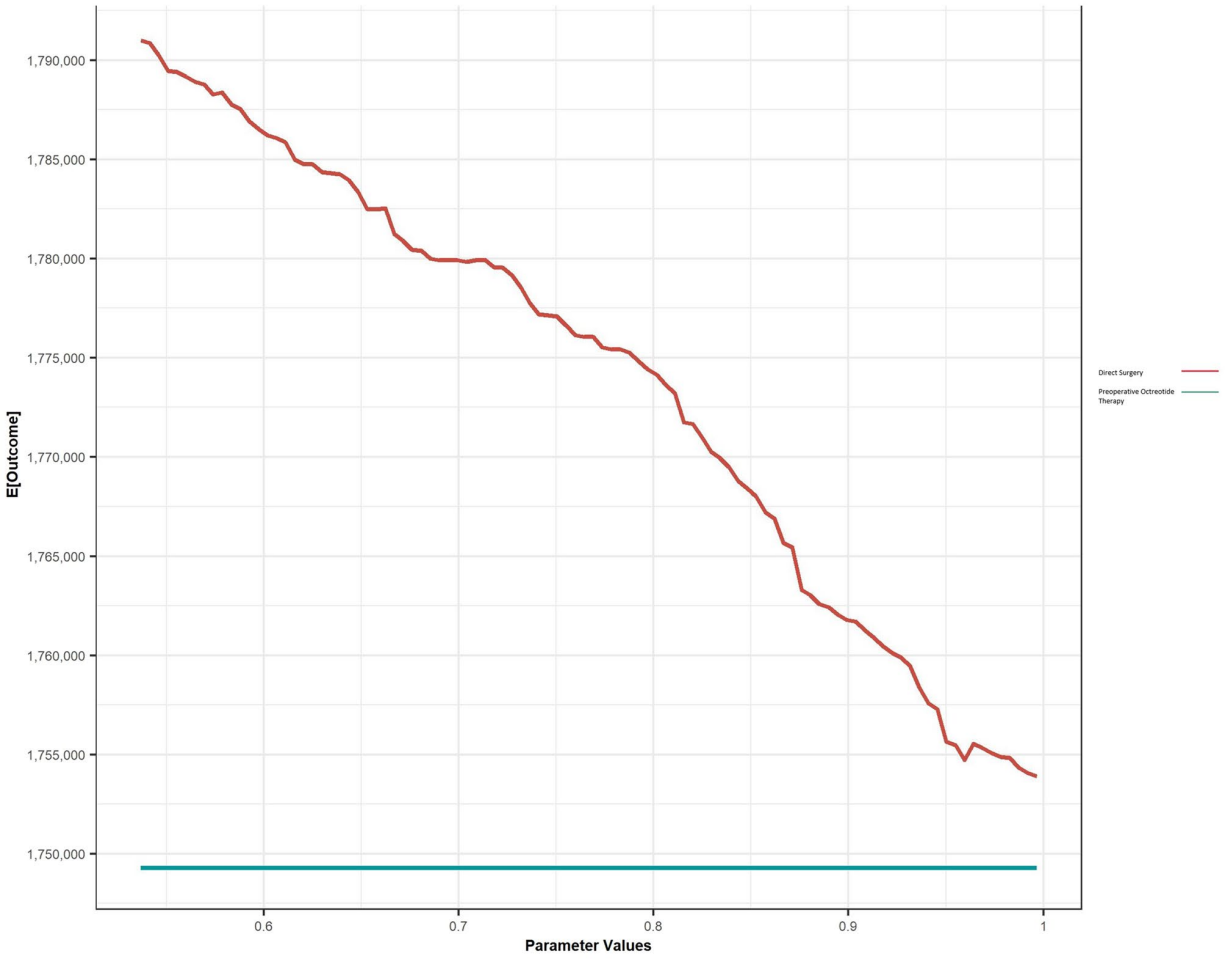

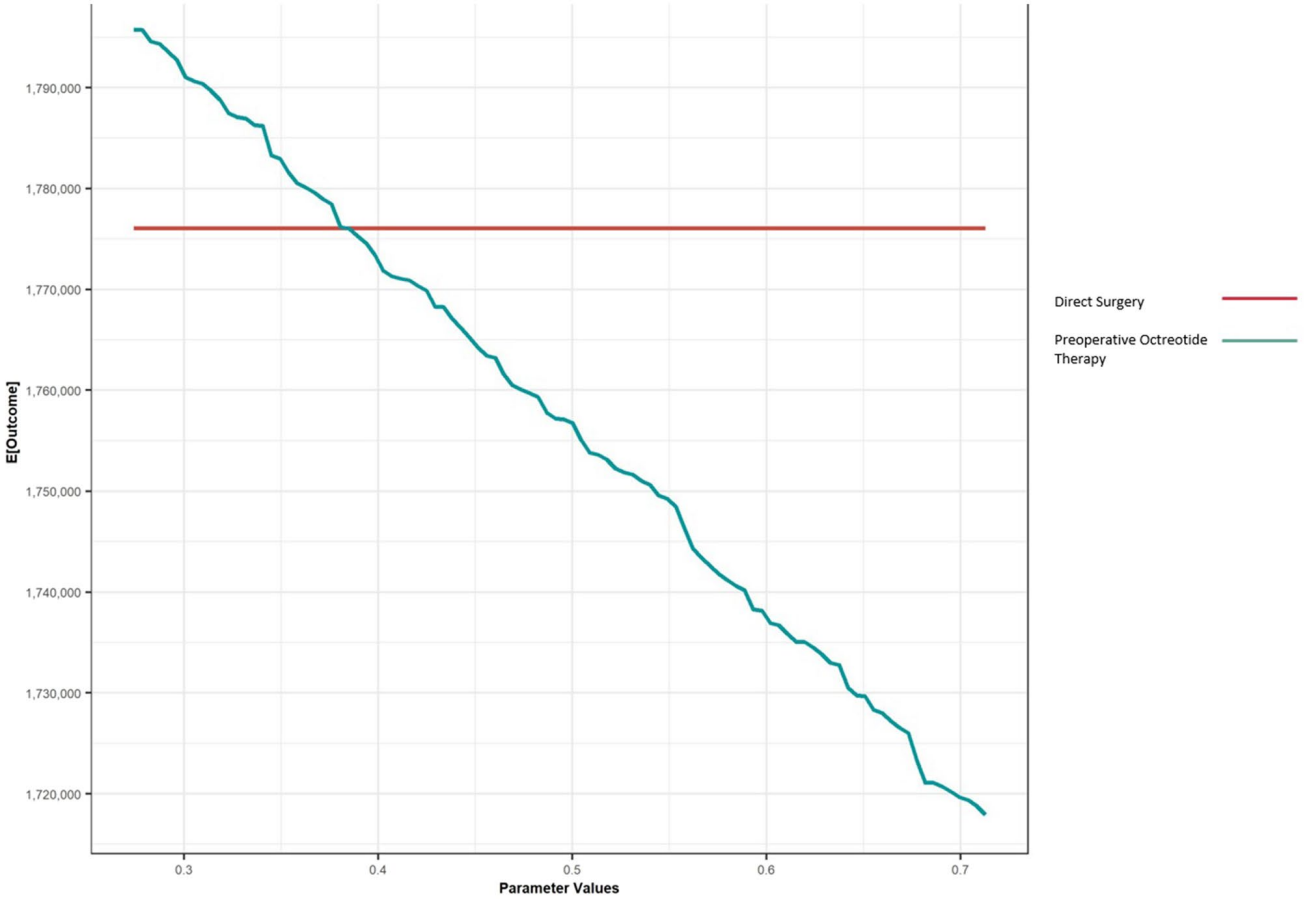
One-way sensitivity analysis. One-way sensitivity analyses determine the effect of changing one variable in the analysis while all others were held constant on the net monetary benefit [99]. **a** Probability of treatment failure after direct surgery. **b** Probability of treatment failure after preoperative octreotide therapy; E (Outcome): Net monetary benefit

One-way sensitivity analyses indicated that results were most sensitive to the probability of treatment failure after direct surgery and preoperative octreotide (Fig. 2 and eFig. 3). The ICER for preoperative octreotide remained above the WTP threshold of $100,000/QALY until probability of treatment failure was less than 38%.

We analyzed clinical scenarios where external beam radiation was initiated following revision surgery instead of gamma knife radiosurgery, the use of gamma knife radiosurgery prior to revision surgery and therapy by tumor subtype (Table 4). Direct surgery remained the preferred strategy in these clinical scenarios. When 3 months of preoperative octreotide therapy was modeled [28], the cost of preoperative octreotide therapy decreased and the health benefits were found to be 0.004 QALYs higher than direct surgery such that, preoperative octreotide therapy became the dominant strategy.

**Table 4:** Results of treatment strategies—second order Monte Carlo simulation of 1000 patients over 1000 iterations (discounted)

Modality	Cost ($)	QALYs	ICER ($/QALY gained)
Scenario 1—post-operative external beam radiation therapy [98]			
Direct surgery	179,670	19.43	–
Preoperative octreotide + surgery	197,745	19.36	Dominated
Scenario 2—gamma-knife radiosurgery following initial surgical failure			
Direct surgery	179,532	19.63	
Preoperative octreotide + surgery	197,743	19.57	Dominated
Scenario 3—patients with microadenoma subtype [26]			
Direct surgery	160,185	19.70	
Preoperative octreotide + surgery	226,921	19.42	Dominated
Scenario 4—patients with macroadenoma subtype [26]			
Direct surgery	184,429	19.43	–
Preoperative octreotide + surgery	195,020	19.41	Dominated
Scenario 5—3 months of preoperative octreotide therapy [28]			
Preoperative octreotide + surgery	183,351	19.377	–
Direct surgery	188,406	19.375	Dominated

An exploratory analysis of second-line pasireotide therapy demonstrated that direct surgery yielded an average of 19.43 QALYs at a cost of $304,066 (eTable 2). The strategy of preoperative octreotide strategy yielded an additional 0.02 QALYs at an additional cost of $8611 with an ICER of $441,116. The preoperative octreotide strategy was not considered a cost-effective strategy at a WTP threshold of $100,000 per QALY. In an analysis of combination pegvisomant and octreotide therapy, direct surgery was found to be the dominant strategy as it yielded lower costs and greater health effects than preoperative octreotide.

**Fig. 3:**
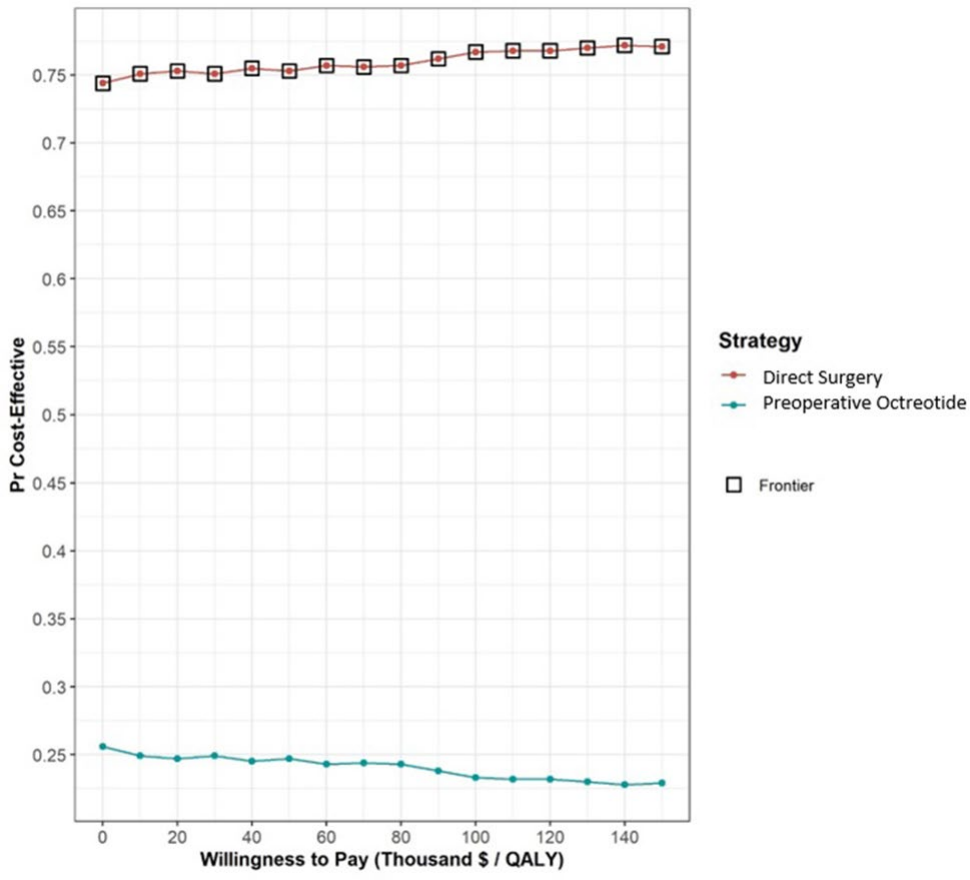
Cost-effectiveness acceptability curves. The acceptability curves are derived from the probabilistic sensitivity analysis and describe the probability that a specific treatment option is cost-effective at different willingness-to-pay thresholds. Different levels of willingness-to-pay (in 1000 US dollar) for a quality-adjusted life year (QALY) gained are defined, and the probabilities of the cost-effectiveness ratio falling below these different thresholds are estimated [99]

When we included the impact of multi-parameter uncertainty, the PSA demonstrated that preoperative octreotide was cost-effective in 23% of 100,000 iterations, given a WTP threshold of $100,000 per QALY gained (Fig. 3).

**Fig. 4:**
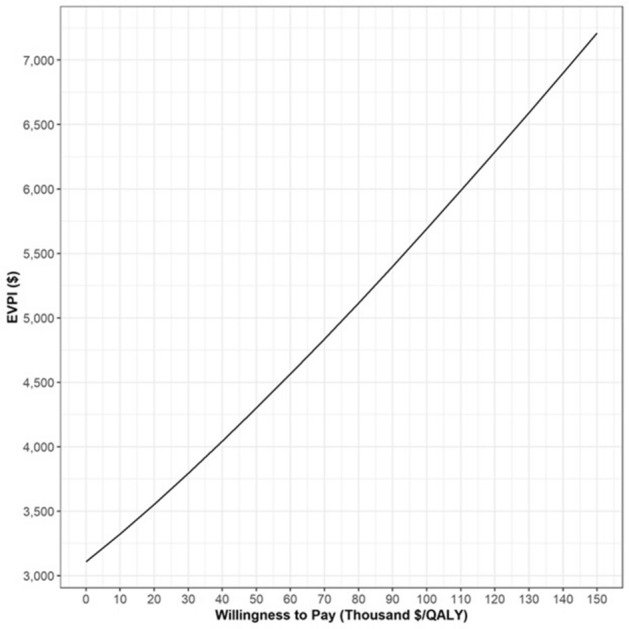
Expected value of perfect information (EVPI) represents the increase in expected value due to parameter uncertainty elimination for different levels of the willingness to pay (thousand $/per quality-adjusted life year (QALY) gained). Interpretation: At a willingness-to-pay threshold of $100,000/QALY the expected value of perfect information is $5700 dollar (per person) and this indicates the maximum amount of net monetary benefit we can gain by collecting perfect information about the model parameters in a population of GH-secreting pituitary adenomas patients

The value of eliminating all uncertainty (i.e., EVPI) started at $3100 per person (Fig. 4). The value gained by acting on perfect information for all parameters rather than on the best evidence currently available steadily increased to a monetized net health benefit of greater than $5700 per person at a WTP of $100,000/QALY. With an estimated 3000 new cases of acromegaly each year in the U.S. [71] and an EVPI of $5700 per case, the findings from this study showed that the EVPI in this patient population is $342,000,000 assuming a 20-year time horizon for the use of these treatment strategies.

The population EVPPI and EVSI quantified the benefit of reducing uncertainty in a health economic model by performing additional studies on the biochemical response rates for the primary treatment strategies at a WTP of $100,000/QALY in the simulated cohort population (eFig. 3). A monetized net health benefit of $435,000 to 500,000 per case was observed for studies of health utility valuation of treatment failure with studies of optimal sample sizes of 200 to 1000. Investigating biochemical response following preoperative octreotide at its optimal sample sizes of 400 to 2000 was associated with monetized net health benefit of $24,000 to 32,500 per case.

## Discussion

Current clinical guidelines do not recommend the routine use of preoperative octreotide therapy in the management of GH-secreting pituitary tumors [14]. However, there remains ongoing controversy regarding the role of preoperative octreotide therapy, as it has been found to result in tumor size reduction and improve surgical remission rates in select prospective studies of acromegaly [26–28, 48, 52, 72, 73]. This decision analytic model demonstrated that 6 months of preoperative octreotide therapy was not cost-effective compared to direct surgery at a WTP threshold of $100,000.

This study builds upon previously published comparisons of preoperative octreotide therapy and direct surgery for acromegaly in several important ways. Informed decision-making based on clinical trials in GH-secreting pituitary adenoma is limited due to small sample size and lack of long term follow up. To date, there have been four randomized clinical trials evaluating preoperative medical therapy and surgery compared to direct surgery, with sample sizes ranging from 39 to 98 participants [26, 28, 48, 73, 74]. Only one clinical trial of 61 patients with GH- secreting adenomas reported up to 5 years of patient follow-up [73]. As such, decision models become an essential tool to understand the impact of treatment strategies on long-term outcomes for this population. A systematic review of the literature demonstrated five previous cost-effectiveness studies of primary surgery vs preoperative drug treatment in patients with acromegaly [75]. Only one study used a Markov model and the model was informed by a retrospective study of 168 cases of a single hospital [76]. In contrast with our study findings, the study reported a higher cost per life year gained in patients that received surgery after preoperative medical therapy compared to direct surgery. The study provided no information on third- and fourth-line therapy for recurrent or persistent disease, uncertainty or sensitivity analyses.

The results of this model were found to be sensitive to the probability of treatment failure after preoperative octreotide therapy and the duration of preoperative octreotide therapy. The results of our model suggested that preoperative octreotide therapy could be considered a cost-effective strategy in the setting of improved biochemical remission (that is, greater than 60% response to primary therapy). The duration of preoperative octreotide therapy has been found to vary across studies from 3 to 6 months [26, 28, 48]. As there is no standardized duration of preoperative octreotide therapy, this model explored duration of preoperative octreotide therapy and found there may be an increase in health benefits associated with preoperative octreotide therapy when limited to 3 months of use prior to surgery.

The WTP threshold of $100,000 used in this study is consistent with published literature as a benchmark for the value of care in the US, yet recent increases in health care spending have called the current threshold into question [77]. A recent classification of health care interventions has suggested that an ICER of < $50,000 per QALY gained should be given a “high level of value” recommendation, while interventions with an ICER of > $150,000 per QALY gained should be given a “low level of value” recommendation [78]. Our model conclusions would recommend preoperative medical therapy for acromegalic patients be classified as a therapy with a low level of value as the ICER was greater than the WTP of $100,000.

Decision models are strengthened by their ability to account for parameter uncertainty in decision-making through probabilistic analysis, which allows decision-makers to quantify the level of confidence in the output of the analysis, measured as the probability that a given strategy is optimal. The study findings suggest that there is a low probability that preoperative octreotide would be cost-effective accounting for parameter uncertainties in the model. However, PSA is only possible to reflect the probability of being cost-effective but does not take into consideration the consequences of being cost-effective or not. Value of information analysis allows integration of the probability of being cost-effective with the consequences of strategies. The EVPI presents the monetized value of the incremental health benefits of eliminating uncertainty from all model parameters over a range of WTP thresholds. Although formal recommendations for when further research should be pursued based on the EVPI are highly context dependent, it has been proposed that authors should not recommend further research for population EVPI values < £250,000 ($163,750 USD). By contrast, authors may recommend further research for population EVPI values > £2 million ($1.3 million USD) [79]. The population EVPI of this model suggests that further research in this realm may be beneficial. Estimates of EVPPI suggested doing additional research on transition probabilities, and health utilities is potentially worthwhile. The EVSI results suggested appropriate sample sizes in future research studies to maximize economic benefit.

### Study limitations and strengths

This study has limitations. First, the analysis is limited by the sample sizes and the low-quality evidence of the included studies used to derive the input parameters. Second, although the model was informed by studies from multiple countries, the analyses were performed for the US setting. As such, the results may not be generalizable to other health care settings as they represent US costing data. Third, recurrences could occur and adjuvant therapy could be obtained at any time during the patient’s lifetime in the model. Fourth, the model inputs were derived from different studies of GH-secreting adenoma populations as it was not possible to use one single source. However, both strategies would have been influenced by these limitations, restricting the effect on the incremental results.

The present study provides a novel and comprehensive evaluation of the treatment approaches for management of acromegaly. The strengths of this study stem from its use of randomized clinical trials from multiple countries to inform the model parameters, the ability to integrate additional therapy for refractory disease, and the exploration of uncertainty to guide further research endeavors. Although this study was limited to US costing data, it provides a framework for decision-making and adds to discussions on the international adoption of this treatment approach.

## Conclusions

Due to conflicting data in the current literature regarding the best approach and sequence of non-surgical and surgical management of GH-secreting adenomas, a synthesis of the evidence on the most effective and safe management was needed to guide decision-making and inform the shift towards individualized patient treatment. From this decision analytic model that integrated the current literature into an evidence-based treatment approach to GH-secreting adenomas, we determined that preoperative octreotide may not be a cost-effective treatment strategy. Further high-quality research into uncertainties in biochemical remission rates following primary therapy and appropriate duration of therapy are warranted to gain further insights and determine conclusively the cost-effectiveness of this treatment strategy.

## Supplementary Information

Below is the link to the electronic supplementary material.

Supplementary file1 (DOCX 2914 kb)

## References

[1] Lorenzoni L, Belloni A, Sassi F (2014). Health-care expenditure and health policy in the USA versus other high-spending OECD countries. Lancet.

[2] OECD (2020) Health spending (indicator). Accessed on 19 Feb 2020

[3] Villwock JA, Villwock M, Deshaies E, Goyal P (2014). Significant increases of pituitary tumors and resections from 1993 to 2011. Int Forum Allergy Rhinol.

[4] Chanson P, Brochier S (2005). Non-functioning pituitary adenomas. J Endocrinol Invest.

[5] Fleseriu M, Bodach ME, Tumialan LM, Bonert V, Oyesiku NM, Patil CG, Litvack Z, Aghi MK, Zada G (2016). Congress of neurological surgeons systematic review and evidence-based guideline for pretreatment endocrine evaluation of patients with nonfunctioning pituitary adenomas. Neurosurgery.

[6] Melmed S (2006). Medical progress: acromegaly. N Engl J Med.

[7] Melmed S (2009). Acromegaly pathogenesis and treatment. J Clin Invest.

[8] Daly AF, Rixhon M, Adam C, Dempegioti A, Tichomirowa MA, Beckers A (2006). High prevalence of pituitary adenomas: a cross-sectional study in the province of Liege, Belgium. J Clin Endocrinol Metab.

[9] Day PF, Loto MG, Glerean M, Picasso MF, Lovazzano S, Giunta DH (2016). Incidence and prevalence of clinically relevant pituitary adenomas: retrospective cohort study in a health management organization in Buenos Aires, Argentina. Arch Endocrinol Metab.

[10] Fernandez A, Karavitaki N, Wass JA (2010). Prevalence of pituitary adenomas: a community-based, cross-sectional study in Banbury. Clin Endocrinol.

[11] Kreutzer J, Vance ML, Lopes MB, Laws ER (2001). Surgical management of GH-secreting pituitary adenomas: an outcome study using modern remission criteria. J Clin Endocrinol Metab.

[12] Melmed S, Kleinberg D, Melmed S, Polonsky PK, Larsen R, Kronenberg H (2016). Pituitary masses and tumors. Williams textbook of endocrinology.

[13] Vilar L, Vilar CF, Lyra R, Lyra R, Naves LA (2017). Acromegaly: clinical features at diagnosis. Pituitary.

[14] Katznelson L, Laws ER, Melmed S, Molitch ME, Murad MH, Utz A, Wass JA, Endocrine S (2014). Acromegaly: an endocrine society clinical practice guideline. J Clin Endocrinol Metab.

[15] Rosario PW (2011). Frequency of acromegaly in adults with diabetes or glucose intolerance and estimated prevalence in the general population. Pituitary.

[16] Sesmilo G, Resmini E, Sambo M, Blanco C, Calvo F, Pazos F, Fernandez-Catalina P, Martinez de Icaya P, Paramo C, Fajardo C, Marazuela M, Alvarez-Escola C, Diez JJ, Perea V (2017). Prevalence of acromegaly in patients with symptoms of sleep apnea. PLoS ONE.

[17] Zahr R, Fleseriu M (2018). Updates in diagnosis and treatment of acromegaly. Eur Endocrinol.

[18] Rokkas T, Pistiolas D, Sechopoulos P, Margantinis G, Koukoulis G (2008). Risk of colorectal neoplasm in patients with acromegaly: a meta-analysis. World J Gastroenterol.

[19] Dworakowska D, Gueorguiev M, Kelly P, Monson JP, Besser GM, Chew SL, Akker SA, Drake WM, Fairclough PD, Grossman AB, Jenkins PJ (2010). Repeated colonoscopic screening of patients with acromegaly: 15-year experience identifies those at risk of new colonic neoplasia and allows for effective screening guidelines. Eur J Endocrinol.

[20] Molitch ME (2017). Diagnosis and treatment of pituitary adenomas: a review. JAMA.

[21] Wolinski K, Czarnywojtek A, Ruchala M (2014). Risk of thyroid nodular disease and thyroid cancer in patients with acromegaly—meta-analysis and systematic review. PLoS ONE.

[22] Dekkers OM, Biermasz NR, Pereira AM, Romijn JA, Vandenbroucke JP (2008). Mortality in acromegaly: a metaanalysis. J Clin Endocrinol Metab.

[23] Holdaway IM, Bolland MJ, Gamble GD (2008). A meta-analysis of the effect of lowering serum levels of GH and IGF-I on mortality in acromegaly. Eur J Endocrinol.

[24] Andries M, Glintborg D, Kvistborg A, Hagen C, Andersen M (2008). A 12-month randomized crossover study on the effects of lanreotide Autogel and octreotide long-acting repeatable on GH and IGF-l in patients with acromegaly. Clin Endocrinol.

[25] Giustina A, Chanson P, Kleinberg D, Bronstein MD, Clemmons DR, Klibanski A, van der Lely AJ, Strasburger CJ, Lamberts SW, Ho KK, Casanueva FF, Melmed S (2014). Expert consensus document: a consensus on the medical treatment of acromegaly. Nat Rev Endocrinol.

[26] Carlsen SM, Lund-Johansen M, Schreiner T, Aanderud S, Johannesen O, Svartberg J, Cooper JG, Hald JK, Fougner SL, Bollerslev J (2008). Preoperative octreotide treatment of acromegaly study, preoperative octreotide treatment in newly diagnosed acromegalic patients with macroadenomas increases cure short-term postoperative rates: a prospective, randomized trial. J Clin Endocrinol Metab.

[27] Fleseriu M, Hoffman AR, Katznelson L, AACE Neuroendocrine and Pituitary Scientific Committee (2015). American Association of Clinical Endocrinologists and American College of Endocrinology disease state clinical review: management of acromegaly patients: what is the role of pre-operative medical therapy?. Endocr Pract.

[28] Shen M, Shou X, Wang Y, Zhang Z, Wu J, Mao Y, Li S, Zhao Y (2010). Effect of presurgical long-acting octreotide treatment in acromegaly patients with invasive pituitary macroadenomas: a prospective randomized study. Endocr J.

[29] Zhang L, Wu X, Yan Y, Qian J, Lu Y, Luo C (2015). Preoperative somatostatin analogs treatment in acromegalic patients with macroadenomas. A meta-analysis. Brain Dev.

[30] Melmed S, Bronstein MD, Chanson P, Klibanski A, Casanueva FF, Wass JAH, Strasburger CJ, Luger A, Clemmons DR, Giustina A (2018). A consensus statement on acromegaly therapeutic outcomes. Nat Rev Endocrinol.

[31] Amato G, Mazziotti G, Rotondi M, Iorio S, Doga M, Sorvillo F, Manganella G, Di Salle F, Giustina A, Carella C (2002). Long-term effects of lanreotide SR and octreotide LAR on tumour shrinkage and GH hypersecretion in patients with previously untreated acromegaly. Clin Endocrinol.

[32] Casagrande A, Bronstein MD, Jallad RS, Moraes AB, Elias PC, Castro M, Czepielewski MA, Boschi A, Ribeiro-Oliveira A, Schweizer JR, Vilar L, Nazato DM, Gadelha MR, Abucham J, All Other Investigators of the Study (2017). Long-term remission of acromegaly after octreotide withdrawal is an uncommon and frequently unsustainable event. Neuroendocrinology.

[33] Ezzat S, Caspar-Bell GM, Chik CL, Denis MC, Domingue ME, Imran SA, Johnson MD, Lochnan HA, Gregoire Nyomba BL, Prebtani A, Ridout R, Ramirez JAR, Van Uum S (2019). Predictive markers for postsurgical medical management of acromegaly: a systematic review and consensus treatment guideline. Endocr Pract.

[34] Tutuncu Y, Berker D, Isik S, Ozuguz U, Akbaba G, Kucukler FK, Aydin Y, Guler S (2012). Comparison of octreotide LAR and lanreotide autogel as post-operative medical treatment in acromegaly. Pituitary.

[35] Sandret L, Maison P, Chanson P (2011). Place of cabergoline in acromegaly: a meta-analysis. J Clin Endocrinol Metab.

[36] Higham CE, Atkinson AB, Aylwin S, Bidlingmaier M, Drake WM, Lewis A, Martin NM, Moyes V, Newell-Price J, Trainer PJ (2012). Effective combination treatment with cabergoline and low-dose pegvisomant in active acromegaly: a prospective clinical trial. J Clin Endocrinol Metab.

[37] Hagen C, Schroeder HD, Hansen S, Hagen C, Andersen M (2009). Temozolomide treatment of a pituitary carcinoma and two pituitary macroadenomas resistant to conventional therapy. Eur J Endocrinol.

[38] Losa M, Mazza E, Terreni MR, McCormack A, Gill AJ, Motta M, Cangi MG, Talarico A, Mortini P, Reni M (2010). Salvage therapy with temozolomide in patients with aggressive or metastatic pituitary adenomas: experience in six cases. Eur J Endocrinol.

[39] Caulley L, Quinn JG, Doyle MA, Alkherayf F, Kilty S, Hunink MGM (2020). Surgical and non-surgical interventions for primary and salvage treatment of growth hormone-secreting pituitary adenomas in adults. Cochrane Database Syst Rev.

[40] Asha MJ, Takami H, Velasquez C, Oswari S, Almeida JP, Zadeh G, Gentili F (2019). Long-term outcomes of transsphenoidal surgery for management of growth hormone-secreting adenomas: single-center results. J Neurosurg.

[41] Heringer LC, de Oliveira MF, Rotta JM, Botelho RV (2016). Effect of repeated transsphenoidal surgery in recurrent or residual pituitary adenomas: a systematic review and meta-analysis. Surg Neurol Int.

[42] Heringer LC, Machado de Lima M, Rotta JM, Botelho RV (2019). Effect of stereotactic radiosurgery on residual or relapsed pituitary adenoma: a systematic review and meta-analysis. World Neurosurg.

[43] Mohammed N, Ding D, Hung YC, Xu Z, Lee CC, Kano H, Martinez-Alvarez R, Martinez-Moreno N, Mathieu D, Kosak M, Cifarelli CP, Katsevman GA, Lunsford LD, Lee Vance M, Sheehan JP (2019). Primary versus postoperative stereotactic radiosurgery for acromegaly: a multicenter matched cohort study. J Neurosurg.

[44] Arias E (2019). United States Life Tables, 2017. Natl Vital Stat Rep.

[45] Ben-Shlomo A, Melmed S (2008). Acromegaly. Endocrinol Metab Clin North Am.

[46] Colao A, Ferone D, Marzullo P, Lombardi G (2004). Systemic complications of acromegaly: epidemiology, pathogenesis, and management. Endocr Rev.

[47] Ament JD, Yang Z, Khatchadourian V, Strong EB, Shahlaie K (2018). Cost-effectiveness of endoscopic versus microscopic transsphenoidal surgery for pituitary adenoma. World Neurosurg.

[48] Mao ZG, Zhu YH, Tang HL, Wang DY, Zhou J, He DS, Lan H, Luo BN, Wang HJ (2010). Preoperative lanreotide treatment in acromegalic patients with macroadenomas increases short-term postoperative cure rates: a prospective, randomised trial. Eur J Endocrinol.

[49] US Bureau of Labor Statistics (2020) Consumer price index: all urban consumers. www.bls.gov/cpi/. Accessed on 5 Nov 2020

[50] Badia X, Trainer P, Biermasz NR, Tiemensma J, Carreno A, Roset M, Forsythe A, Webb SM (2018). Mapping AcroQoL scores to EQ-5D to obtain utility values for patients with acromegaly. J Med Econ.

[51] Global Burden of Disease Study 2017 (GBD 2017) (2018) Disability weights. In: G.B.o.D.C. Network (ed) Institute for Health Metrics and Evaluation (IHME), Seattle

[52] Li ZQ, Quan Z, Tian HL, Cheng M (2012). Preoperative lanreotide treatment improves outcome in patients with acromegaly resulting from invasive pituitary macroadenoma. J Int Med Res.

[53] Li T, Alkherayf F, Malcolm J, Arnaout A, Lochnan H, Keely E, Agbi C, Doyle MA (2018). 107 - Clinical characteristics and outcomes of patients treated for acromegaly at a Tertiary Care Hospital. Can J Diabetes.

[54] Sanders GD, Neumann PJ, Basu A, Brock DW, Feeny D, Krahn M, Kuntz KM, Meltzer DO, Owens DK, Prosser LA, Salomon JA, Sculpher MJ, Trikalinos TA, Russell LB, Siegel JE, Ganiats TG (2016). Recommendations for conduct, methodological practices, and reporting of cost-effectiveness analyses: second panel on cost-effectiveness in health and medicine. JAMA.

[55] Gadelha MR, Bronstein MD, Brue T, Coculescu M, Fleseriu M, Guitelman M, Pronin V, Raverot G, Shimon I, Lievre KK, Fleck J, Aout M, Pedroncelli AM, Colao A, Pasireotide CSG (2014). Pasireotide versus continued treatment with octreotide or lanreotide in patients with inadequately controlled acromegaly (PAOLA): a randomised, phase 3 trial. Lancet Diabetes Endocrinol.

[56] Truong HL, Nellesen D, Ludlam WH, Neary MP (2014). Budget impact of pasireotide for the treatment of Cushing’s disease, a rare endocrine disorder associated with considerable comorbidities. J Med Econ.

[57] Neggers SJ, Franck SE, de Rooij FW, Dallenga AH, Poublon RM, Feelders RA, Janssen JA, Buchfelder M, Hofland LJ, Jorgensen JO, van der Lely AJ (2014). Long-term efficacy and safety of pegvisomant in combination with long-acting somatostatin analogs in acromegaly. J Clin Endocrinol Metab.

[58] Caulley L, Hunink MG, Randolph GW, Shin JJ (2020). Evidence-based medicine in otolaryngology, part XI: modeling and analysis to support decisions. Otolaryngol Head Neck Surg.

[59] Retel VP, Grutters JP, van Harten WH, Joore MA (2013). Value of research and value of development in early assessments of new medical technologies. Value Health.

[60] Claxton K, Sculpher M, Drummond M (2002). A rational framework for decision making by the National Institute for Clinical Excellence (NICE). Lancet.

[61] Claxton KP, Sculpher MJ (2006). Using value of information analysis to prioritise health research: some lessons from recent UK experience. Pharmacoeconomics.

[62] Oostenbrink JB, Al MJ, Oppe M, Rutten-van Molken MP (2008). Expected value of perfect information: an empirical example of reducing decision uncertainty by conducting additional research. Value Health.

[63] Minelli C, Baio G (2015). Value of information: a tool to improve research prioritization and reduce waste. PLoS Med.

[64] Schmidt C (2010). Researchers consider value-of-information theory for selecting trials. J Natl Cancer Inst.

[65] Claxton K, Neumann PJ, Araki S, Weinstein MC (2001). Bayesian value-of-information analysis. An application to a policy model of Alzheimer’s disease. Int J Technol Assess Health Care.

[66] Appendix 1, What is the expected value of perfect information in reducing uncertainty surrounding the cost-effectiveness of systemic treatment in patients with confirmed carcinoma of unknown primary and no clinical features fitting a recognised syndrome? https://www.ncbi.nlm.nih.gov/books/NBK82153/ (2010 2022)

[67] Jalal H, Alarid-Escudero F (2018). A Gaussian approximation approach for value of information analysis. Med Decis Making.

[68] Alarid-Escudero F, Krijkamp EM, Pechlivanoglou P, Jalal H, Kao SZ, Yang A, Enns EA (2019). A need for change! A coding framework for improving transparency in decision modelling. Pharmacoeconomics.

[69] Krijkamp EM, Alarid-Escudero F, Enns EA, Jalal HJ, Hunink MGM, Pechlivanoglou P (2018). Microsimulation modeling for health decision sciences using R: a tutorial. Med Decis Making.

[70] Husereau D, Drummond M, Augustovski F, de Bekker-Grob E, Briggs A, Carswell C, Caulley L, Chaiyakunapruk N, Greenberg D, Loder E, Mauskopf J, Mullins C, Petrou S, Pwu R, Staniszewska S (2022). CHEERS 2022 ISPOR Good Research Practices Task Force. Consolidated Health Economic Evaluation Reporting Standards 2022 (CHEERS 2022) statement: updated reporting guidance for health economic evaluations. BMJ.

[71] Broder MS, Chang E, Cherepanov D, Neary MP, Ludlam WH (2016). Incidence and prevalence of acromegaly in the United States: a claims-based analysis. Endocr Pract.

[72] Bolanowski M, Zgliczynski W, Sowinski J, Baldys-Waligorska A, Bednarek-Tupikowska G, Witek P, Zielinski G, Liebert W, Sieminska L, Andrysiak-Mamos E, Marek B, Kajdaniuk D, Malicka J, Rosiek V, Jawiarczyk-Przybylowska A, Investigators CB (2020). Therapeutic effect of presurgical treatment with longacting octreotide (Sandostatin(R) LAR(R)) in patients with acromegaly. Endokrynol Pol.

[73] Fougner SL, Bollerslev J, Svartberg J, Oksnes M, Cooper J, Carlsen SM (2014). Preoperative octreotide treatment of acromegaly: long-term results of a randomised controlled trial. Eur J Endocrinol.

[74] Li A, Liu W, Cao P, Zheng Y, Bu Z, Zhou T (2017). Endoscopic versus microscopic transsphenoidal surgery in the treatment of pituitary adenoma: a systematic review and meta-analysis. World Neurosurg.

[75] Leonart LP, Borba HHL, Ferreira VL, Riveros BS, Pontarolo R (2018). Cost-effectiveness of acromegaly treatments: a systematic review. Pituitary.

[76] Duan L, Huang M, Yan H, Zhang Y, Gu F (2015). Cost-effectiveness analysis of two therapeutic schemes in the treatment of acromegaly: a retrospective study of 168 cases. J Endocrinol Invest.

[77] Neumann PJ, Cohen JT, Weinstein MC (2014). Updating cost-effectiveness–the curious resilience of the $50,000-per-QALY threshold. N Engl J Med.

[78] Anderson JL, Heidenreich PA, Barnett PG, Creager MA, Fonarow GC, Gibbons RJ, Halperin JL, Hlatky MA, Jacobs AK, Mark DB, Masoudi FA, Peterson ED, Shaw LJ (2014). ACC/AHA statement on cost/value methodology in clinical practice guidelines and performance measures: a report of the American College of Cardiology/American Heart Association task force on performance measures and task force on practice guidelines. J Am Coll Cardiol.

[79] Thorn J, Coast J, Andronis L (2016). Interpretation of the expected value of perfect information and research recommendations: a systematic review and empirical investigation. Med Decis Making.

[80] Lavrentaki A, Paluzzi A, Wass JA, Karavitaki N (2017). Epidemiology of acromegaly: review of population studies. Pituitary.

[81] Fahlbusch R, Kleinberg D, Biller B, Bonert V, Buchfelder M, Cappabianca P, Carmichael J, Chandler W, Colao A, George A, Klibanski A, Knopp E, Kreutzer J, Kundurti N, Lesser M, Mamelak A, Pivonello R, Post K, Swearingen B, Vance ML, Barkan A (2017). Surgical debulking of pituitary adenomas improves responsiveness to octreotide lar in the treatment of acromegaly. Pituitary.

[82] Colao A, Cappabianca P, Caron P, De Menis E, Farrall AJ, Gadelha MR, Hmissi A, Rees A, Reincke M, Safari M, T’Sjoen G, Bouterfa H, Cuneo RC (2009). Octreotide LAR vs. surgery in newly diagnosed patients with acromegaly: a randomized, open-label, multicentre study. Clin Endocrinol.

[83] Wilson TJ, McKean EL, Barkan AL, Chandler WF, Sullivan SE (2013). Repeat endoscopic transsphenoidal surgery for acromegaly: remission and complications. Pituitary.

[84] Jagannathan J, Sheehan JP, Pouratian N, Laws ER, Steiner L, Vance ML (2008). Gamma knife radiosurgery for acromegaly: outcomes after failed transsphenoidal surgery. Neurosurgery.

[85] Feenstra J, de Herder WW, ten Have SM, van den Beld AW, Feelders RA, Janssen JA, van der Lely AJ (2005). Combined therapy with somatostatin analogues and weekly pegvisomant in active acromegaly. Lancet.

[86] McCormack A, Dekkers OM, Petersenn S, Popovic V, Trouillas J, Raverot G, Burman P, ESE Survey Collaborators (2018). Treatment of aggressive pituitary tumours and carcinomas: results of a European Society of Endocrinology (ESE) survey. Eur J Endocrinol.

[87] Ammirati M, Wei L, Ciric I (2013). Short-term outcome of endoscopic versus microscopic pituitary adenoma surgery: a systematic review and meta-analysis. J Neurol Neurosurg Psychiatry.

[88] Rudmik L, Starreveld YP, Vandergrift WA, Banglawala SM, Soler ZM (2015). Cost-effectiveness of the endoscopic versus microscopic approach for pituitary adenoma resection. Laryngoscope.

[89] Karaca Z, Tanriverdi F, Elbuken G, Cakir I, Donmez H, Selcuklu A, Durak AC, Dokmetas HS, Colak R, Unluhizarci K, Kelestimur F (2011). Comparison of primary octreotide-lar and surgical treatment in newly diagnosed patients with acromegaly. Clin Endocrinol.

[90] Cho DY, Tsao M, Lee WY, Chang CS (2006). Socioeconomic costs of open surgery and gamma knife radiosurgery for benign cranial base tumors. Neurosurgery.

[91] Tiemensma J, Kaptein AA, Pereira AM, Smit JW, Romijn JA, Biermasz NR (2011). Affected illness perceptions and the association with impaired quality of life in patients with long-term remission of acromegaly. J Clin Endocrinol Metab.

[92] Lobatto DJ, Zamanipoor Najafabadi AH, de Vries F, Andela CD, van den Hout WB, Pereira AM, Peul WC, Vliet Vlieland TPM, van Furth WR, Biermasz NR (2019). Toward value based health care in pituitary surgery: application of a comprehensive outcome set in perioperative care. Eur J Endocrinol.

[93] GBD 2019 Diseases and Injuries Collaborators (2020). Global burden of 369 diseases and injuries in 204 countries and territories, 1990–2019: a systematic analysis for the Global Burden of Disease Study 2019. Lancet.

[94] Oosmanally N, Paul JE, Zanation AM, Ewend MG, Senior BA, Ebert CS (2011). Comparative analysis of cost of endoscopic endonasal minimally invasive and sublabial-transseptal approaches to the pituitary. Int Forum Allergy Rhinol.

[95] Ayyagari R, Neary M, Li S, Rokito A, Yang H, Xie J, Benson AB (2017). Comparing the cost of treatment with octreotide long-acting release versus lanreotide in patients with metastatic gastrointestinal neuroendocrine tumors. Am Health Drug Benefits.

[96] Moore DJ, Adi Y, Connock MJ, Bayliss S (2009). Clinical effectiveness and cost-effectiveness of pegvisomant for the treatment of acromegaly: a systematic review and economic evaluation. BMC Endocr Disord.

[97] Wasserfallen JB, Ostermann S, Leyvraz S, Stupp R (2005). Cost of temozolomide therapy and global care for recurrent malignant gliomas followed until death. Neuro-Oncology.

[98] Biermasz NR, van Dulken H, Roelfsema F (2000). Long-term follow-up results of postoperative radiotherapy in 36 patients with acromegaly. J Clin Endocrinol Metab.

[99] Otero HJ, Rybicki FJ, Greenberg D, Neumann PJ (2008). Twenty years of cost-effectiveness analysis in medical imaging: are we improving?. Radiology.

