# Supplementary material for: Cost-effectiveness of direct surgery versus preoperative octreotide therapy for growth-hormone secreting pituitary adenomas
Source: Pituitary. 2022 Aug 27;25(6):868–81. doi: 10.1007/s11102-022-01270-8 (PMC9675692; doi:10.1007/s11102-022-01270-8)
Supplement: Supplementary file 1 — Supplementary file1 (DOCX 2914 kb) [file 11102_2022_1270_MOESM1_ESM.docx]

Appendix

eFigure 1. Hazard of treatment failure over time by treatment strategy

eFigure 2. Markov trace for decision analytic model

eFigure 3. Additional one-way sensitivity analyses

eFigure 4. Expected value of partially perfect information and expected value of sample information

eTable 1. Model validation

eTable 2. Exploratory scenario analyses

eFigure 1. Hazard of treatment failure over time by treatment strategy

1. Surgery alone[1-5]


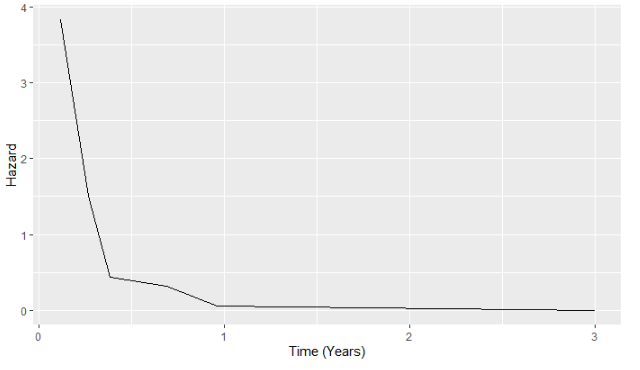


1. Preoperative medical therapy and surgery alone [2, 4, 6]


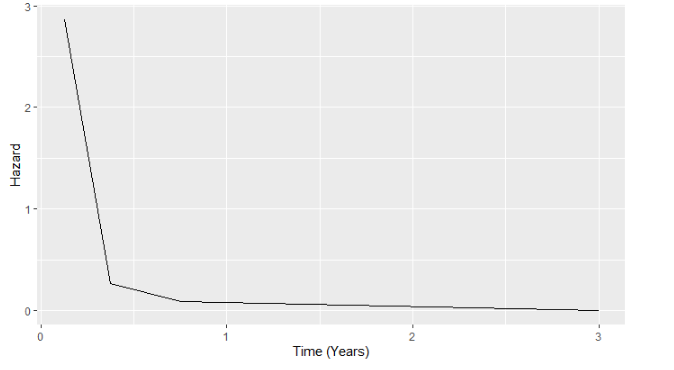


eFigure 2. Markov trace for the decision analytic model. The Markov trace illustrates the first-order Monte Carlo simulation of 100,000 patients with growth hormone-secreting pituitary adenomas as they move between health states in accordance with the specified transition probabilities. A) Direct Surgery B) Preoperative Octreotide Therapy

A)


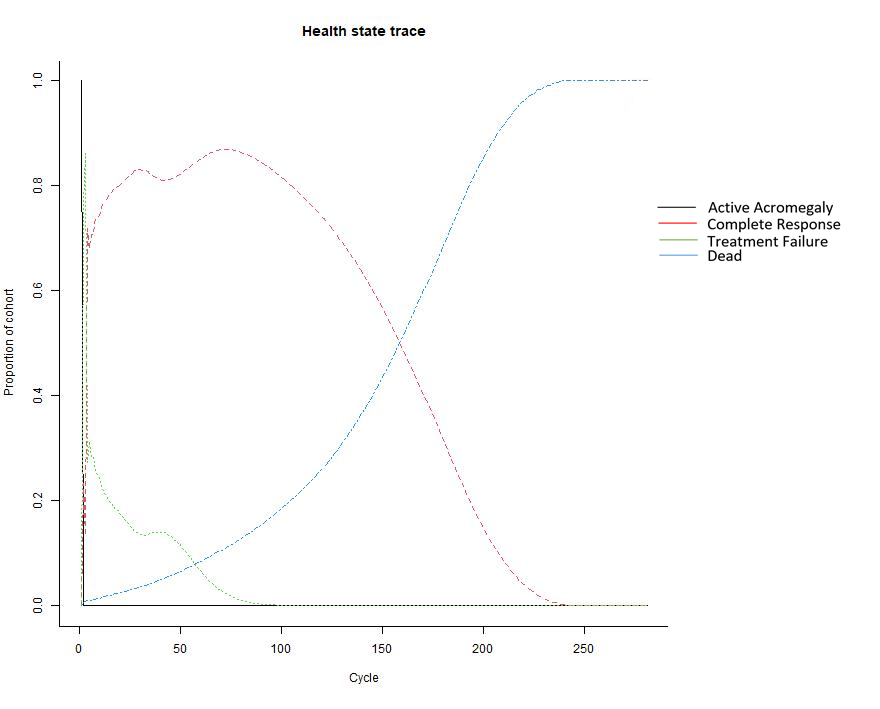


B)


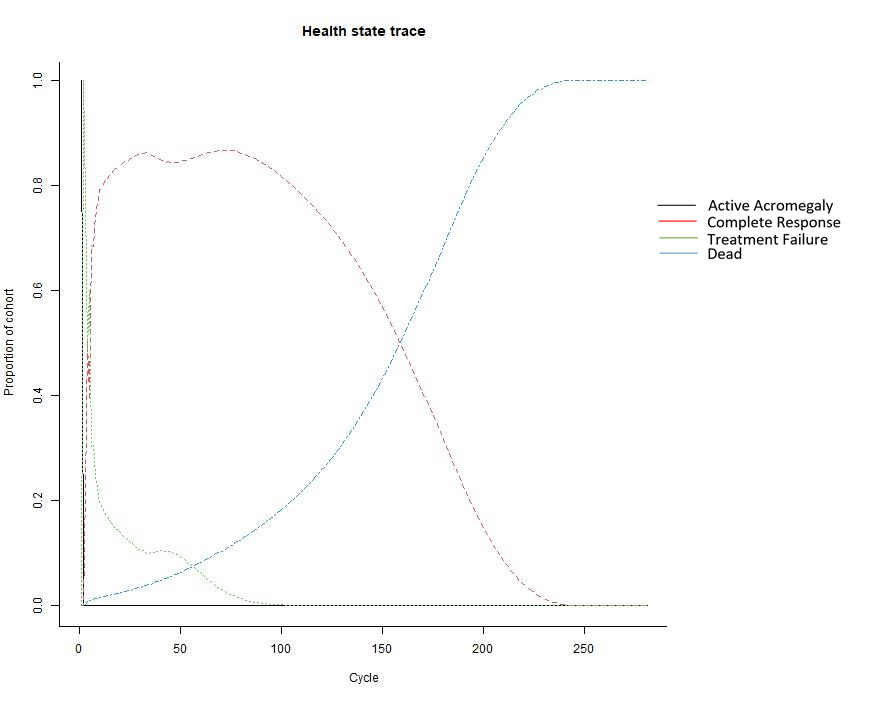


eFigure 3. Additional one-way sensitivity analyses; E(Outcomes): Net Monetary Benefit


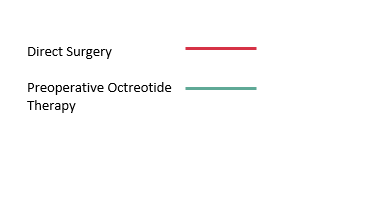


A) Probabilities; i-Probability of treatment failure after adjuvant radiosurgery, ii-Probability of hematoma after surgery, iii-Probability of treatment failure after post-operative cabergoline and pegvisomant therapy, iv – Probability of treatment failure after post-operative octreotide and cabergoline, v- Probability of treatment failure after revision surgery, vi-Probability of treatment failure after salvage therapy, vii-Probability of transient diabetes insipidus after surgery, viii- Probability of complications after octreotide therapy, ix-Probability of complications after adjuvant radiosurgery, x-Probability of death after surgery, xi- Probability of cerebrospinal fluid leak after surgery, xii- Probability of persistent diabetes insipidus after surgery, xiii-Probability of hypopituitarism after surgery, xiv-Probability of visual loss after surgery

ii

i


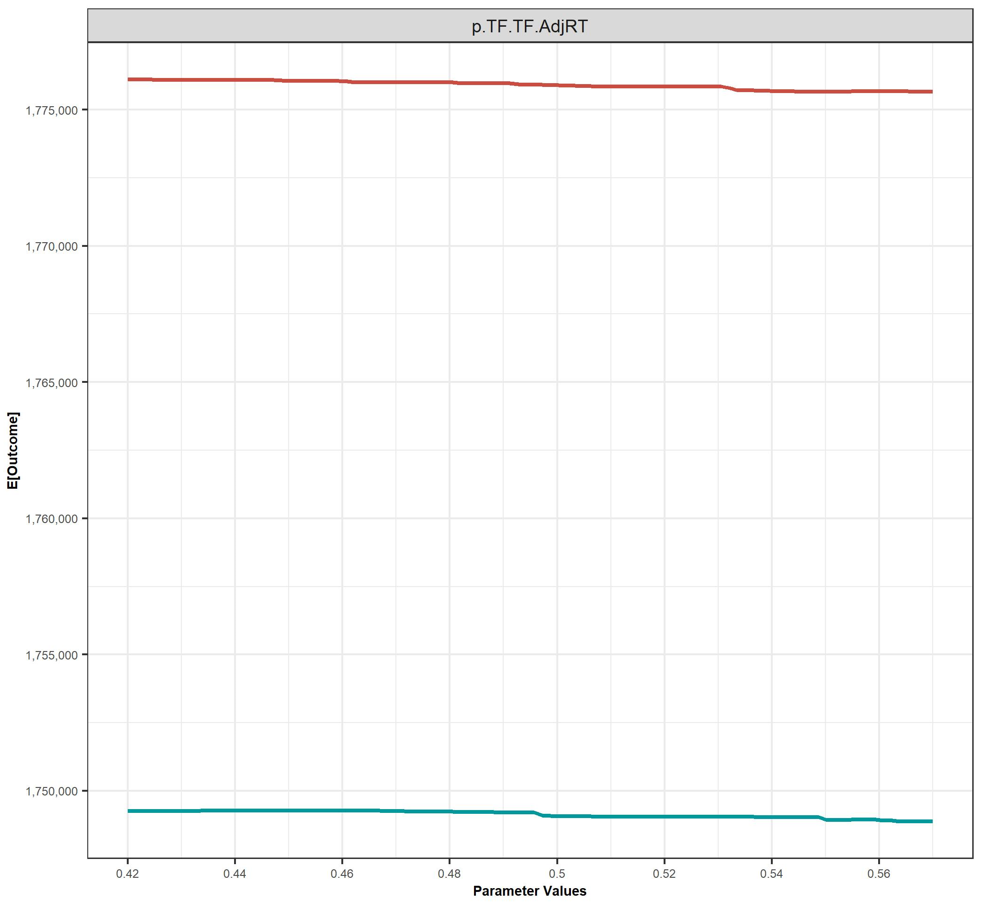

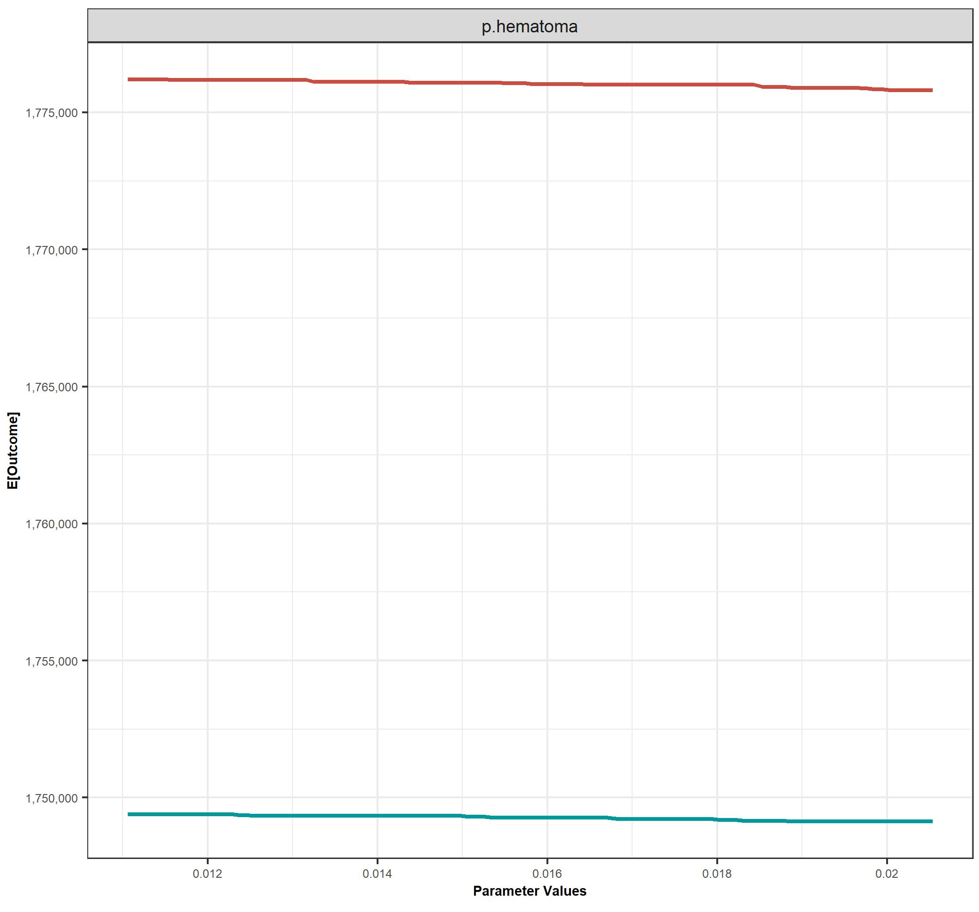


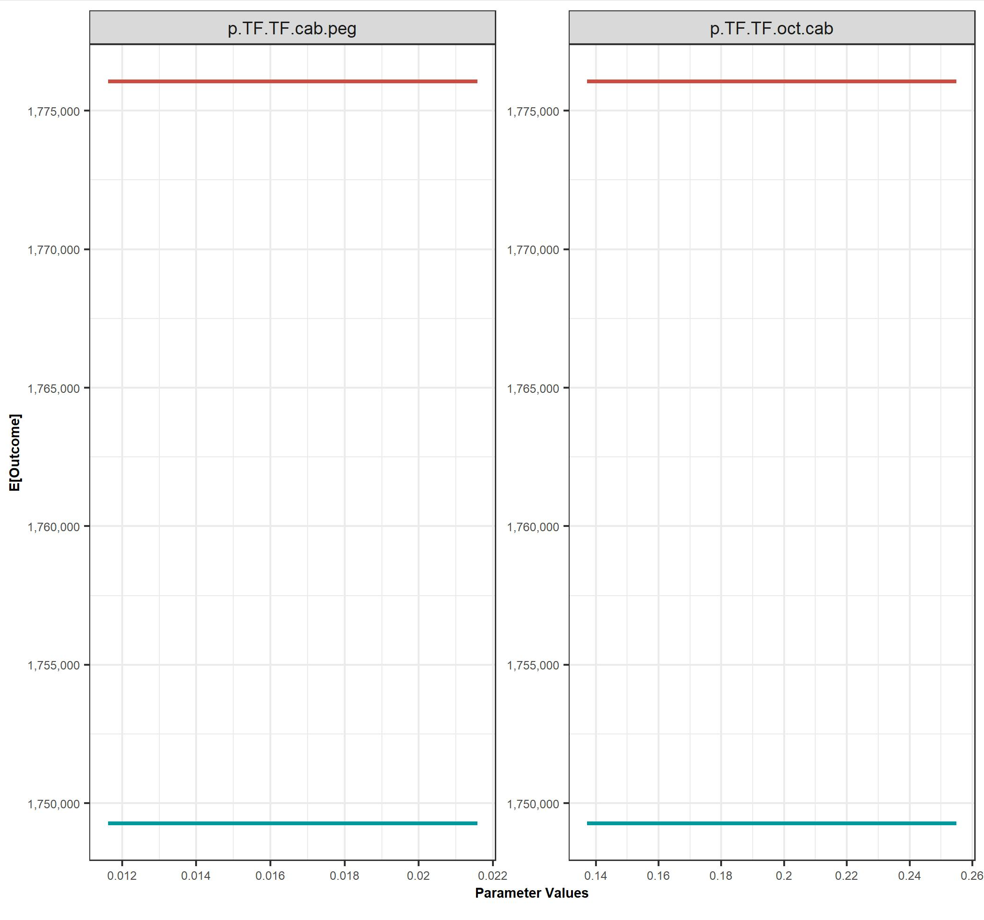

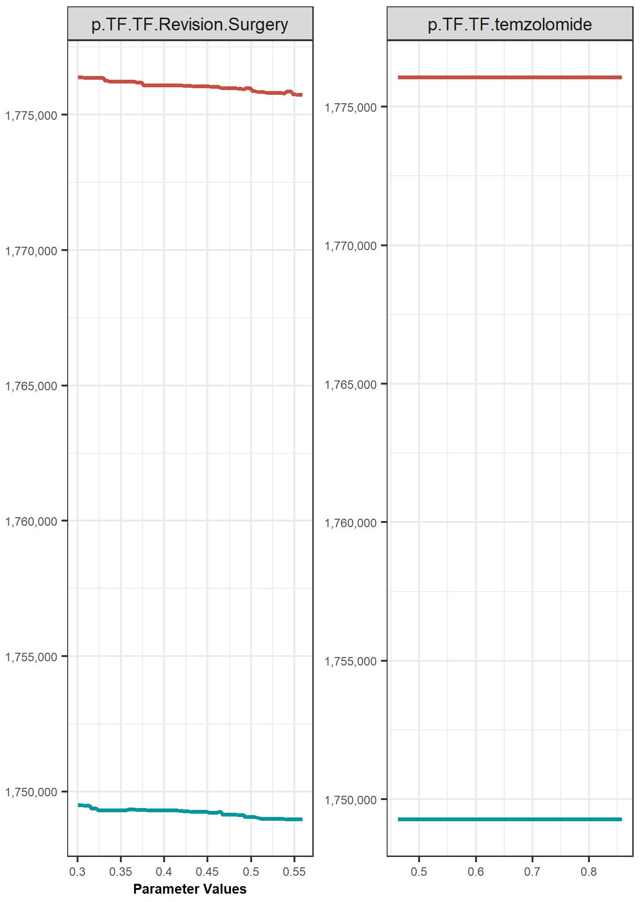

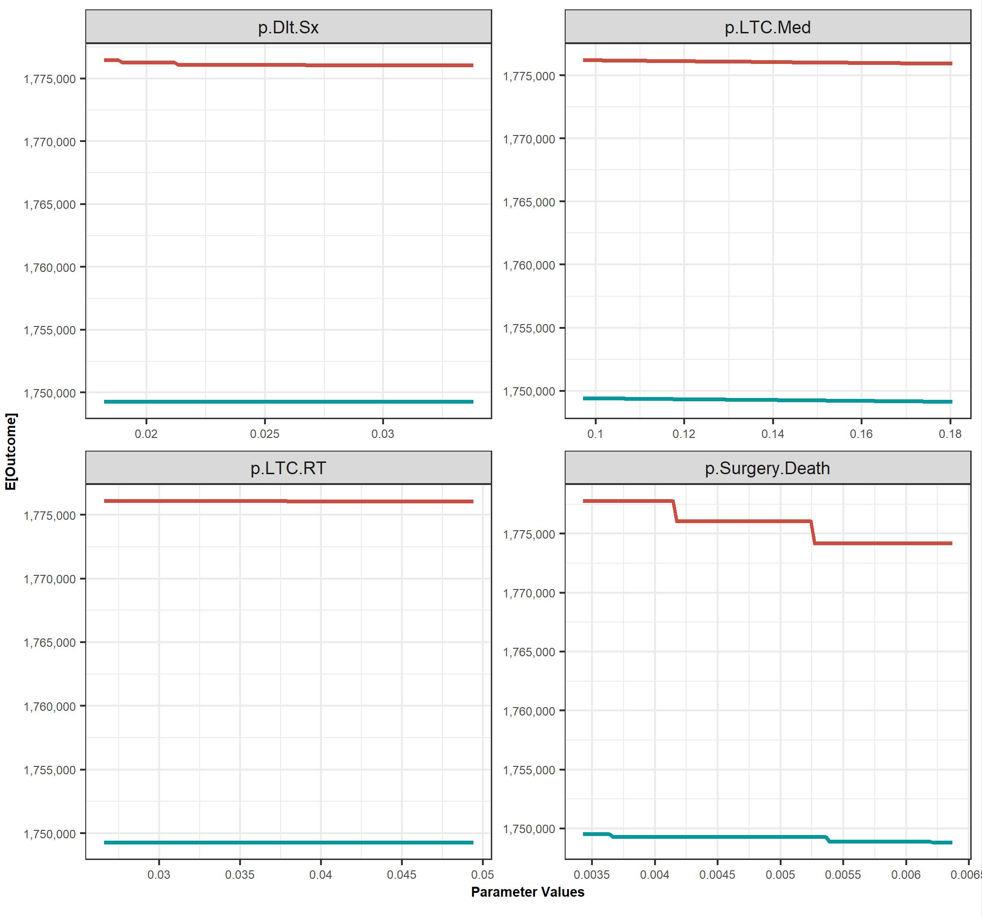


iv

iii

**E[Outcome]**

vi

v

x

ix

viii

vii


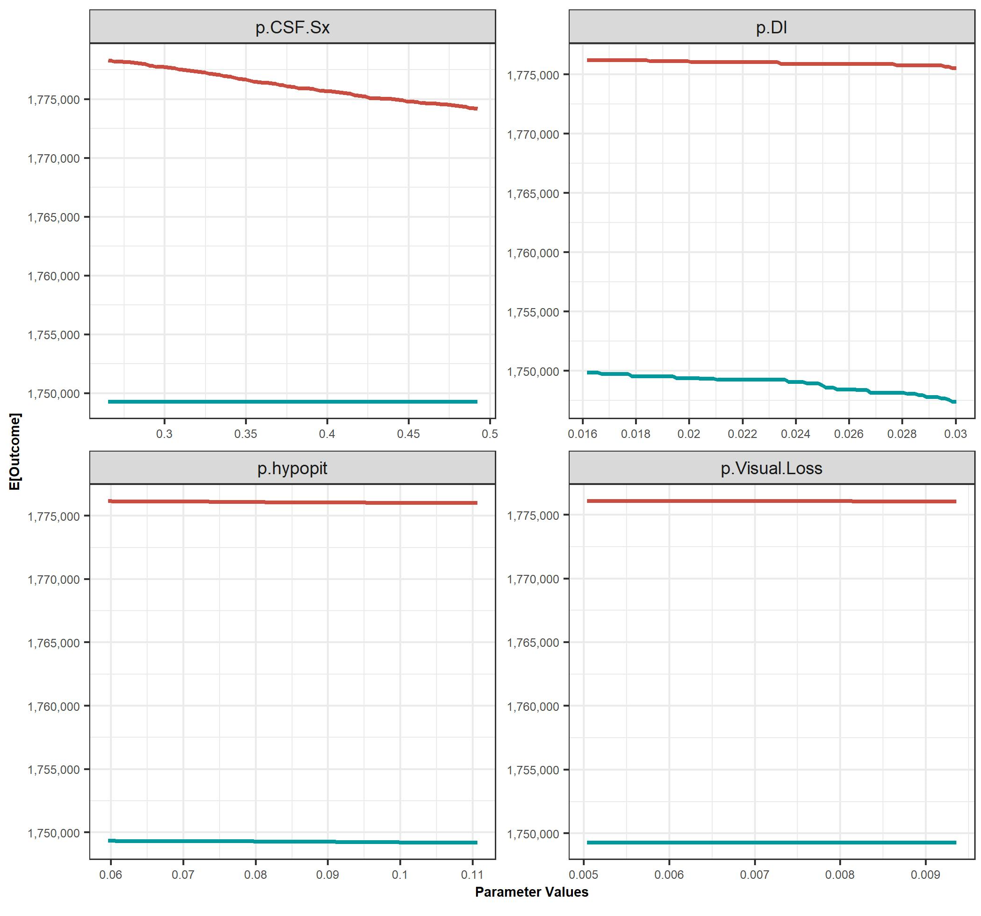


xiv

xiii

xii

xi

B) Cost; i-Cost of cabergoline, ii-Cost of pegvisomant, iii-Cost of cerebrospinal fluid leak after surgery, iv- Cost of complications after adjuvant radiosurgery, v- Cost of adjuvant gamma knife radiosurgery, vi- Cost of salvage therapy, vii- Cost of persistent diabetes insipidus, viii – Cost of transient diabetes insipidus, ix – Cost of hematoma after surgery, x- Cost of vision loss after surgery, xi- Cost of hypopituitarism after surgery, xii-Cost of complications after octreotide therapy


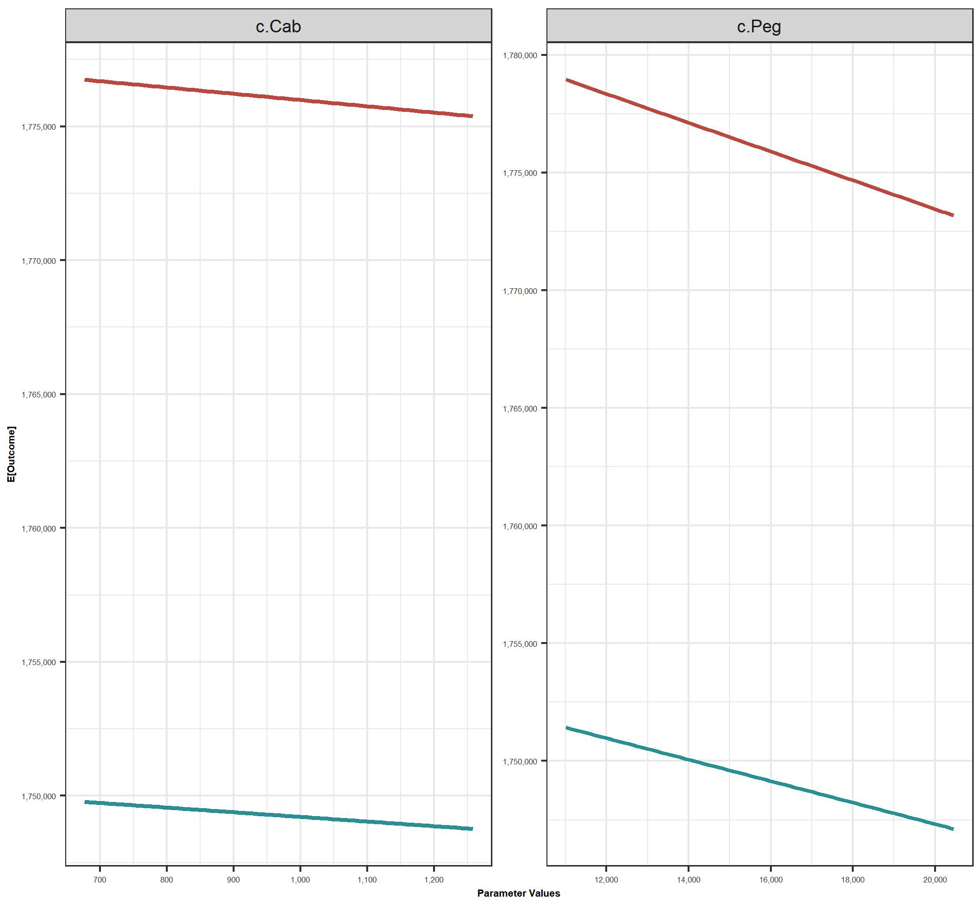


ii

i


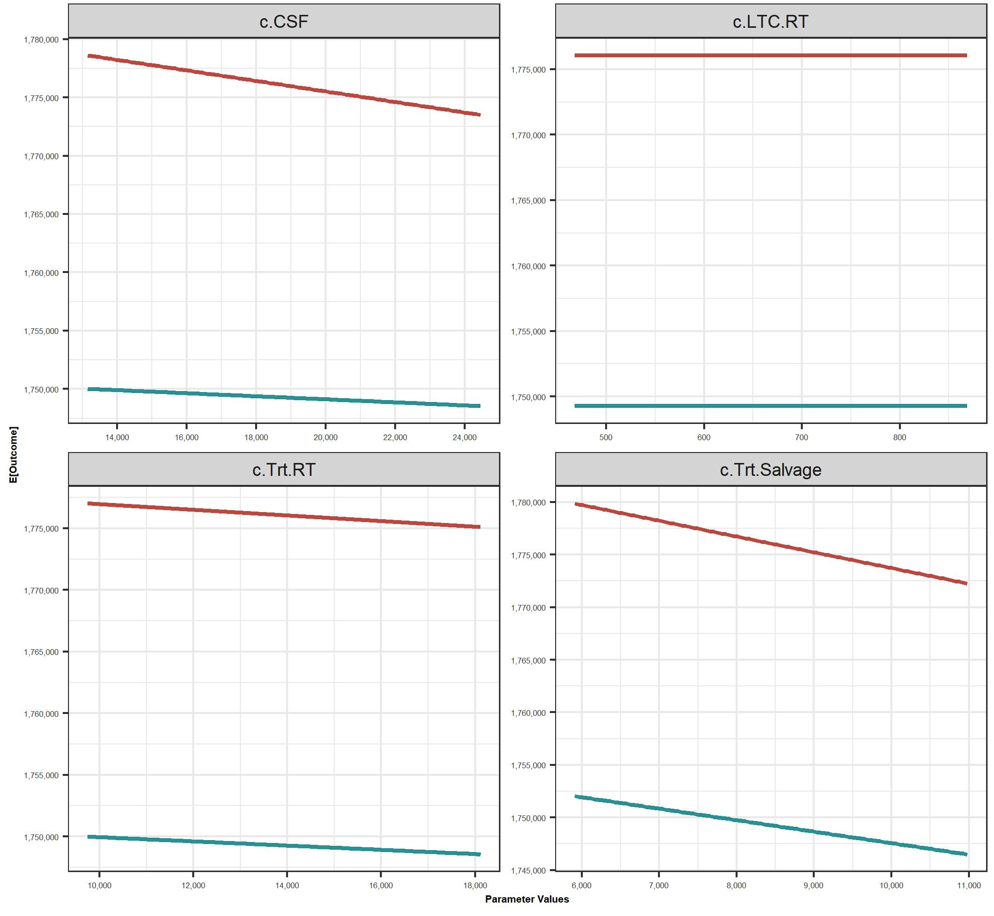


vi

v

iv

iii


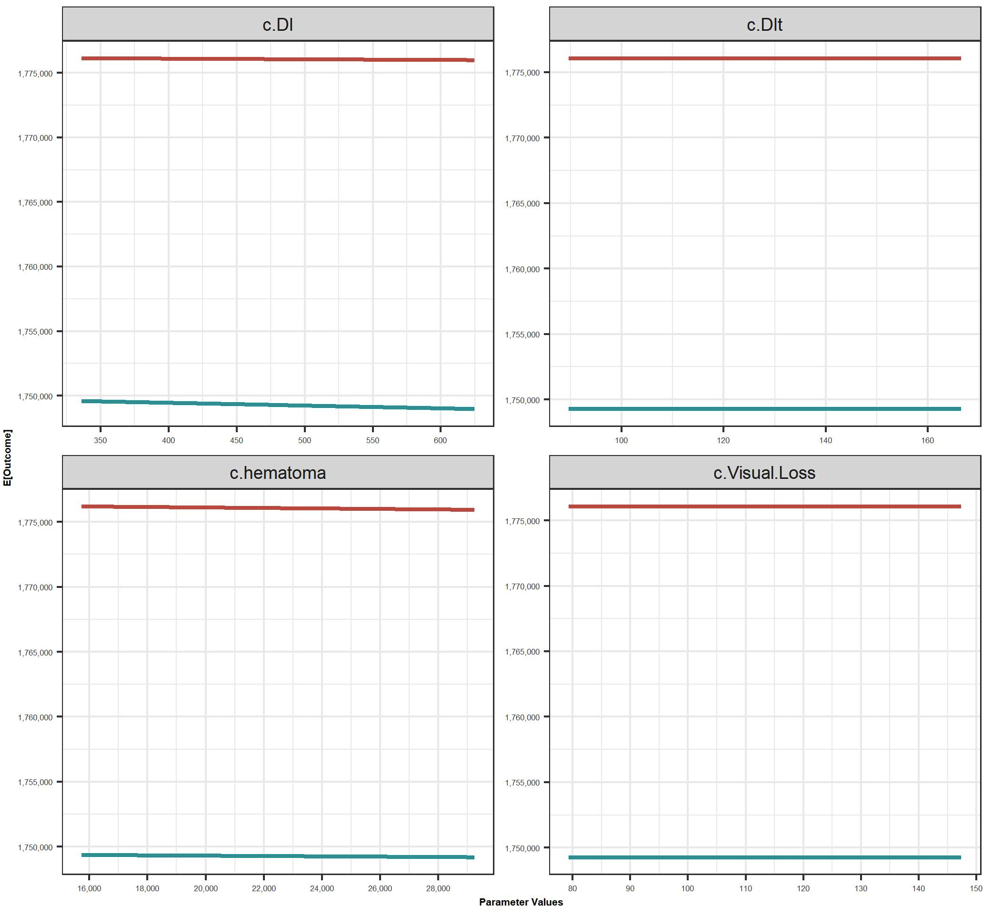


x

ix

viii

vii


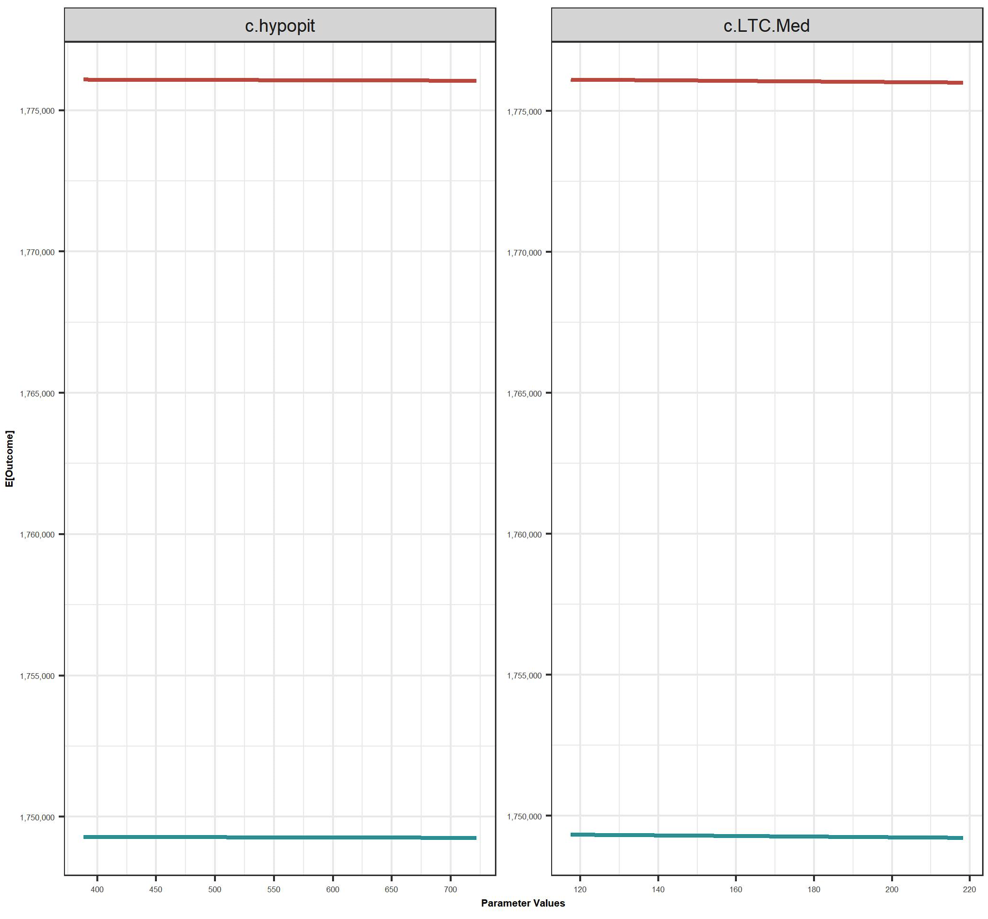


xii

xi

C) Utility Weights: i - Cerebrospinal fluid leak after surgery, ii – Transient diabetes insipidus after surgery, iii – Persistent diabetes insipidus after surgery, iv – complications after adjuvant radiosurgery, v- Hematoma after surgery, vi-Hypopituitarism after surgery, vii- Complications after medical therapy, viii – Visual loss after surgery, ix – Odds ratio of cerebrospinal fluid leak after preoperative octreotide therapy compared to direct surgery, x- Odds ratio of diabetes insipidus after preoperative octreotide therapy compared to direct surgery, xi – discount rate, xii- Standardized mortality rate for patients with untreated acromegaly


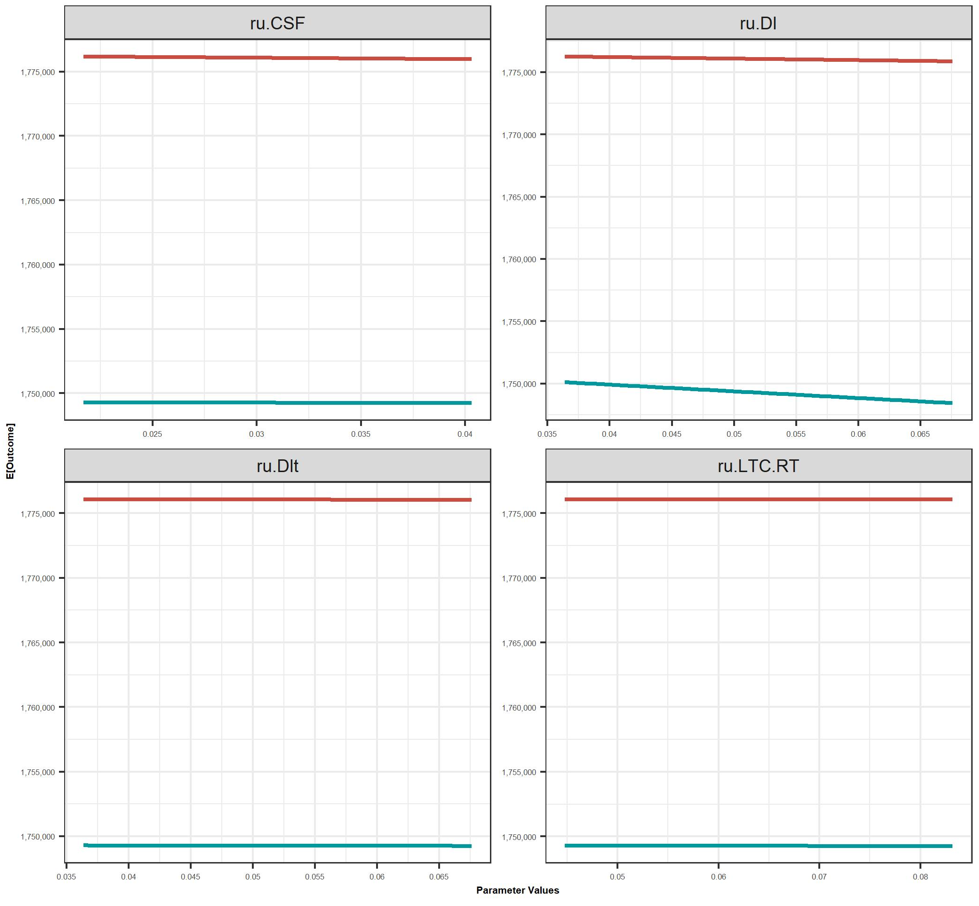


-

-

-

-

-

-

-

-

-

-

-

-

-

-

-

-

-

-

-

-

-

-

ii

i

iv

iii


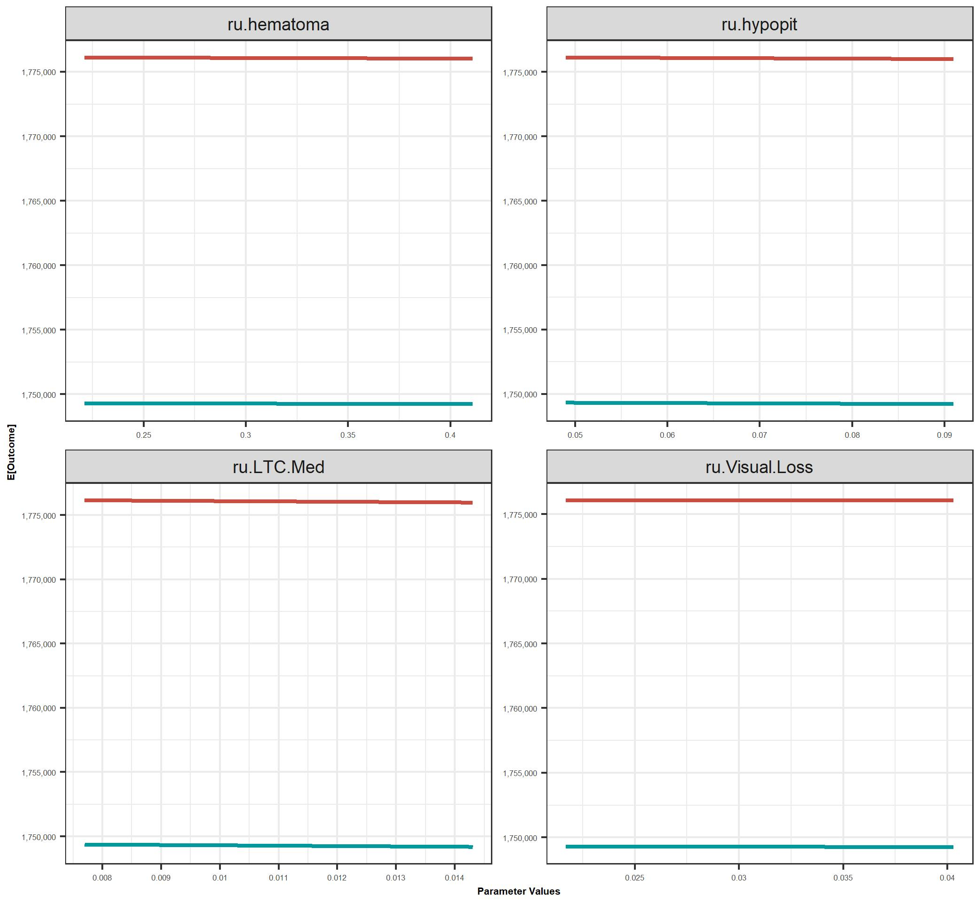


-

-

-

-

-

-

-

-

-

-

-

-

-

-

-

-

-

-

-

-

v

viii

vii

vi


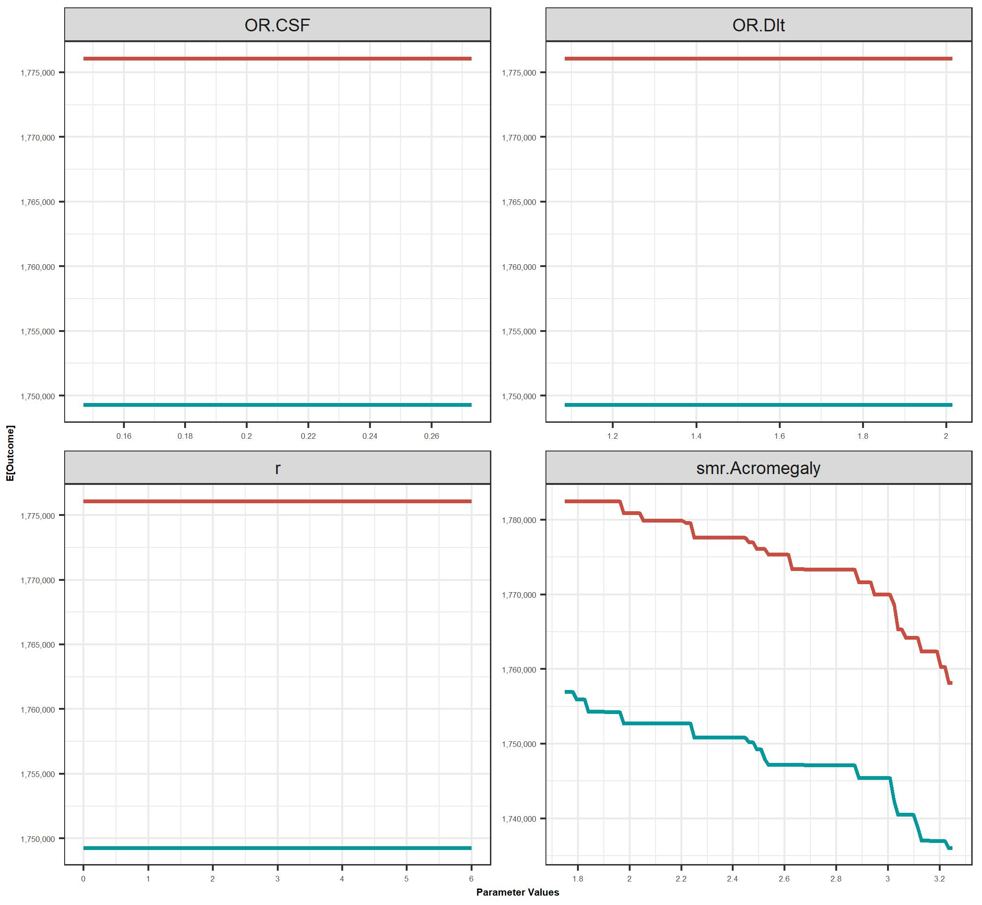


xii

xi

x

ix

eFigure 4. Population expected value of partial perfect information and sample information by parameter investigated depending on sample size of the future study.

1. Health utility of treatment failure for growth-hormone secreting pituitary adenomas
2. Probability of treatment failure following pre-treatment and surgery
3.
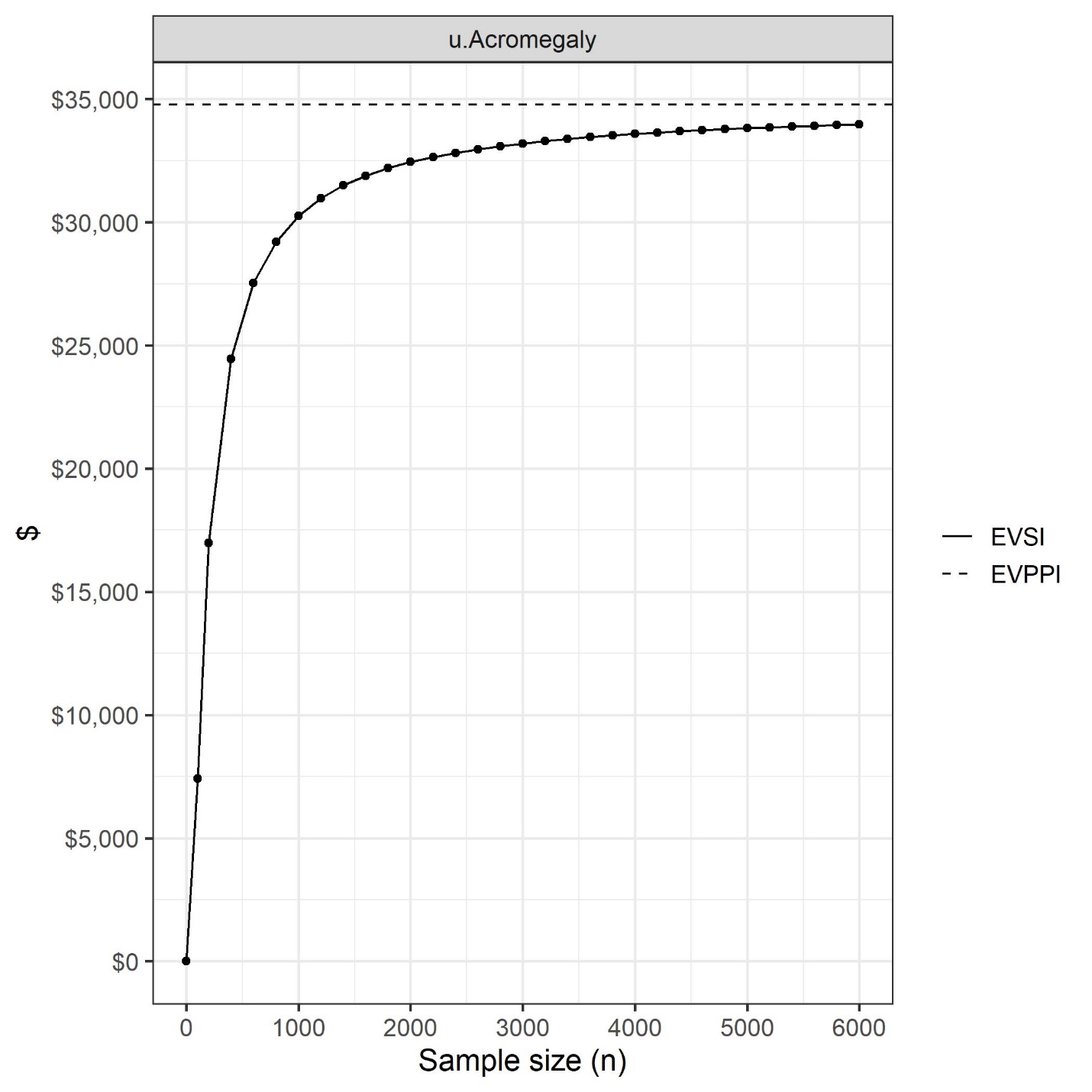

4.
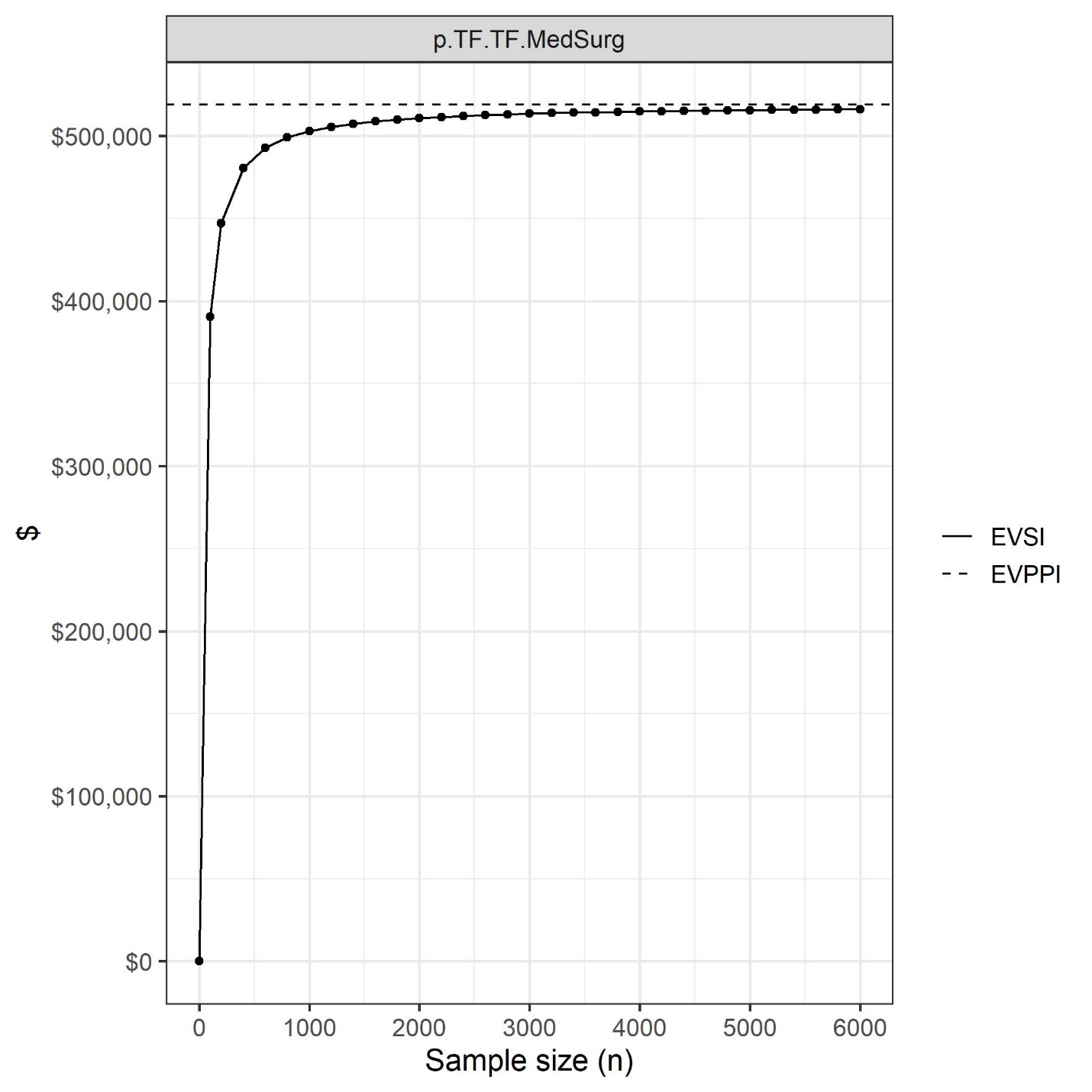


eTable 1. Model Validation

1. Complete response at 18 months after standard treatment (that is, direct surgery alone)

|  | Complete Response (% [95% Confidence Interval]) | Reference |
| --- | --- | --- |
| Model | 69.3% (66.3 – 72.1%) | - |
| Data | 63.9% (46.2 – 79.2%) | [7] |

b) Need for adjuvant therapy in standard treatment (that is, direct surgery)

|  | Proportion of Patients (% [95% Confidence Interval]) | Reference |
| --- | --- | --- |
| Revision Surgery |  |  |
| Model | 33.5% (30.6 – 36.5%) | - |
| Data | 28.9% (16.4 – 44.3%) | [8] |
| Radiation Therapy |  |  |
| Model | 31.5% (28.6 – 34.5%) | - |
| Data | 30.9% (24.1 – 38.3%) | [9] |

eTable 2. Results of Treatment Strategies – Second Order Monte Carlo Simulation of 1000 patients over 1000 iterations (Discounted)

| Modality | Cost ($) | QALYs | ICER ($/QALY gained) |
| --- | --- | --- | --- |
| *Scenario 1 –Second Line Pasireotide Therapy* | 304,065 | 19.43 |  |
| *Direct Surgery* | 312,676 | 19.45 | 441,116 |
| *Preoperative Octreotide + Surgery* |  |  |  |
| *Scenario 2 –Combination therapy Pegvisomant and Octreotide* |  |  |  |
| *Direct Surgery* | 191,760 | 19.63 |  |
| *Preoperative Octreotide + Surgery* | 206,870 | 19.56 | Dominated |

**REFERENCES**

[1] A. Colao, P. Cappabianca, P. Caron, E. De Menis, A.J. Farrall, M.R. Gadelha, A. Hmissi, A. Rees, M. Reincke, M. Safari, G. T'Sjoen, H. Bouterfa, R.C. Cuneo, Octreotide LAR vs. surgery in newly diagnosed patients with acromegaly: a randomized, open-label, multicentre study, Clin Endocrinol (Oxf) 70(5) (2009) 757-68.

[2] S.M. Carlsen, M. Lund-Johansen, T. Schreiner, S. Aanderud, O. Johannesen, J. Svartberg, J.G. Cooper, J.K. Hald, S.L. Fougner, J. Bollerslev, g. Preoperative Octreotide Treatment of Acromegaly study, Preoperative octreotide treatment in newly diagnosed acromegalic patients with macroadenomas increases cure short-term postoperative rates: a prospective, randomized trial, J Clin Endocrinol Metab 93(8) (2008) 2984-90.

[3] R. Fahlbusch, D. Kleinberg, B. Biller, V. Bonert, M. Buchfelder, P. Cappabianca, J. Carmichael, W. Chandler, A. Colao, A. George, A. Klibanski, E. Knopp, J. Kreutzer, N. Kundurti, M. Lesser, A. Mamelak, R. Pivonello, K. Post, B. Swearingen, M.L. Vance, A. Barkan, Surgical debulking of pituitary adenomas improves responsiveness to octreotide lar in the treatment of acromegaly, Pituitary 20(6) (2017) 668-675.

[4] S.L. Fougner, J. Bollerslev, J. Svartberg, M. Oksnes, J. Cooper, S.M. Carlsen, Preoperative octreotide treatment of acromegaly: long-term results of a randomised controlled trial, Eur J Endocrinol 171(2) (2014) 229-35.

[5] Z.Q. Li, Z. Quan, H.L. Tian, M. Cheng, Preoperative lanreotide treatment improves outcome in patients with acromegaly resulting from invasive pituitary macroadenoma, J Int Med Res 40(2) (2012) 517-24.

[6] M. Shen, X. Shou, Y. Wang, Z. Zhang, J. Wu, Y. Mao, S. Li, Y. Zhao, Effect of presurgical long-acting octreotide treatment in acromegaly patients with invasive pituitary macroadenomas: a prospective randomized study, Endocr J 57(12) (2010) 1035-44.

[7] Y. Tutuncu, D. Berker, S. Isik, U. Ozuguz, G. Akbaba, F.K. Kucukler, Y. Aydin, S. Guler, Comparison of octreotide LAR and lanreotide autogel as post-operative medical treatment in acromegaly, Pituitary 15(3) (2012) 398-404.

[8] T. Li, F. Alkherayf, J. Malcolm, A. Arnaout, H. Lochnan, E. Keely, C. Agbi, M.A. Doyle, Characteristics and Outcomes of Patients Treated for Acromegaly at a Tertiary Care Center, (2021).

[9] D.H. Davis, E.R. Laws, Jr., D.M. Ilstrup, J.K. Speed, M. Caruso, E.G. Shaw, C.F. Abboud, B.W. Scheithauer, L.M. Root, C. Schleck, Results of surgical treatment for growth hormone-secreting pituitary adenomas, J Neurosurg 79(1) (1993) 70-5.
